# reChIP-seq reveals widespread bivalency of H3K4me3 and H3K27me3 in CD4^+^ memory T cells

**DOI:** 10.1038/ncomms12514

**Published:** 2016-08-17

**Authors:** Sarah Kinkley, Johannes Helmuth, Julia K. Polansky, Ilona Dunkel, Gilles Gasparoni, Sebastian Fröhler, Wei Chen, Jörn Walter, Alf Hamann, Ho-Ryun Chung

**Affiliations:** 1 Otto-Warburg-Laboratory: Epigenomics, Max Planck Institute for Molecular Genetics, Ihnestrasse 63-73, 14195 Berlin, Germany; 2 Experimental Rheumatology, German Rheumatism Research Center Berlin, Charitéplatz 1, 10117 Berlin, Germany; 3 The Department of Genetics and Epigenetics, University of Saarland, Campus A2.4 66123 Saarbrücken, Germany; 4 The Laboratory of Functional Genomics and Systems Biology, Max Delbruck Centrum for Molecular Medicine, Robert-Rössle-Strasse 10, 13125 Berlin, Germany

## Abstract

The combinatorial action of co-localizing chromatin modifications and regulators determines chromatin structure and function. However, identifying co-localizing chromatin features in a high-throughput manner remains a technical challenge. Here we describe a novel reChIP-seq approach and tailored bioinformatic analysis tool, normR that allows for the sequential enrichment and detection of co-localizing DNA-associated proteins in an unbiased and genome-wide manner. We illustrate the utility of the reChIP-seq method and normR by identifying H3K4me3 or H3K27me3 bivalently modified nucleosomes in primary human CD4^+^ memory T cells. We unravel widespread bivalency at hypomethylated CpG-islands coinciding with inactive promoters of developmental regulators. reChIP-seq additionally uncovered heterogeneous bivalency in the population, which was undetectable by intersecting H3K4me3 and H3K27me3 ChIP-seq tracks. Finally, we provide evidence that bivalency is established and stabilized by an interplay between the genome and epigenome. Our reChIP-seq approach augments conventional ChIP-seq and is broadly applicable to unravel combinatorial modes of action.

## Abstract (web summary)

Co-localizing chromatin modifications and regulators can exert a combinatorial effect on chromatin structure and function. Here the authors describe reChIP-seq and normR to identify co-localizing proteins in an unbiased genome-wide manner.

## Graphical abstract

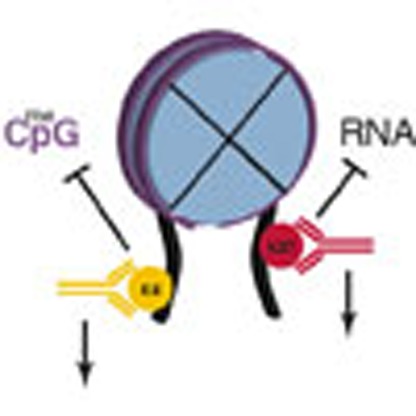

Eukaryotic genomes are packaged into chromatin, whose basic repeating unit is the nucleosome, consisting of two copies of each core histone, H2A, H2B, H3 and H4 with ∼147 base pairs of DNA^1^,^2^. The structure and function of chromatin, determines cell-type-specific gene expression patterns and cellular identity, which is in part achieved through post-translational histone modifications. The true complexity of this histone language is thought to be delivered through combinatorial histone modifications^3^,^4^.

The co-occurrence of bivalent H3K4me3 and H3K27me3 histone modifications, was discovered in embryonic stem cells (ESCs)^5^, at developmental genes leading to the hypothesis that bivalency maintains a poised state^5^,^6^. However, the presence of bivalent domains in differentiated cells, such as pyramidal neurons^7^ and T cells^8^,^9^ argues that bivalency serves a more common function. The concept of H3K4me3 and rH3K27me3 bivalency has been met with criticism for several reasons: (i) H3K4me3 and H3K27me3 are not usually present on the same peptide^10^; (ii) *in vitro* methylation of H3K27 by polycomb repressive complex 2, is inhibited by the presence of H3K4me3 (refs 11, 12); (iii) vice versa the trimethylation of H3K4 by KMT2 complexes is inhibited by the presence of H3K27me3 (ref. 13); and (iv) due to a lack of direct experimental evidence of the co-occurrence of H3K4me3 and H3K27me3.

Currently, intersection of H3K4me3 and H3K27me3 ChIP-seq tracks is the sole method to detect bivalent domains genome-wide. However, co-enrichment is not sufficient to establish whether H3K4me3 and H3K27me3 are indeed present on the same nucleosome and therefore co-localize. They may be present at the same loci albeit in different cells and/or alleles. To address this criticism, reChIP qPCR approaches^14^,^15^,^16^,^17^ have been used to validate co-localization of H3K4me3 and H3K27me3 (ref. 5). While instructive, a major caveat of these reChIP experiments is the inefficient capture of the second co-localizing mark, due to harsh elution steps after the first ChIP. As a consequence, large amounts of input chromatin are required to obtain sufficient DNA for sequencing^18^. Furthermore, the adverse effect of these harsh elution steps on chromatin epitopes and thereby on the second ChIP remains unclear. Consequently, the few genome-wide reChIP studies that have been conducted, have predominantly utilized tagged transgenes^19^,^20^, with one exception where this approach was applied to endogenous targets^21^.

Here, we describe a highly versatile and efficient reChIP followed by sequencing (reChIP-seq) approach that utilizes a much milder elution of bound chromatin through competition with specific peptides. This in turn generates a chromatin template suitable for a sequential precipitation of a second antigen, allowing for the identification of endogenous co-existing histone modifications on single nucleosomes genome-wide. Existing bioinformatic approaches^22^,^23^,^24^,^25^,^26^ are not well suited to call enrichment in (re)ChIPs with low signal-to-noise ratio because they fail to account for the effect of enrichment on the overall read statistics (Supplementary Note 1). Therefore we have developed a novel bioinformatic approach called normR (Supplementary Software, available also at https://bioconductor.org/packages/normr), which models reChIP-seq and control primary ChIP-seq read counts to identify regions enriched for co-localizing histone modifications.

As a proof of principle, we used reChIP-seq and normR to generate a genome-wide map of bivalency in primary human CD4^+^ central memory T cells (TCMs). We explore these unprecedented data and show that (i) H3K4me3 and H3K27me3 co-exist on single nucleosomes; (ii) that bivalency occurs at hypomethylated CpG-rich and inactive transcriptional start sites (TSSs); (iii) that bivalent targets in TCMs predominantly mark developmental regulators that largely overlap with bivalent domains in human ESCs; (iv) that 14% of bivalent TSSs identified by classical co-enrichment of H3K4me3 and H3K27me3 are not enriched in reChIP-seq, indicating that they are pseudo bivalent; (v) that the co-enrichment approach additionally fails to detect up to 60% of bivalent promoters; and (vi) finally, that bivalency and/or H3K4me3 is an inherent feature of hypomethylated CpG-rich sequences, which appears to be stable during development including in the germline.

## Results

### The reChIP-seq and normR method

Conventional ChIP-seq approaches are not designed to discern the co-localization of distinct proteins or histone modifications on the same DNA template. Therefore, studies aimed at identifying combinatorial histone features genome-wide are challenged by the lack of an appropriate experimental approach.

**Figure 1:**
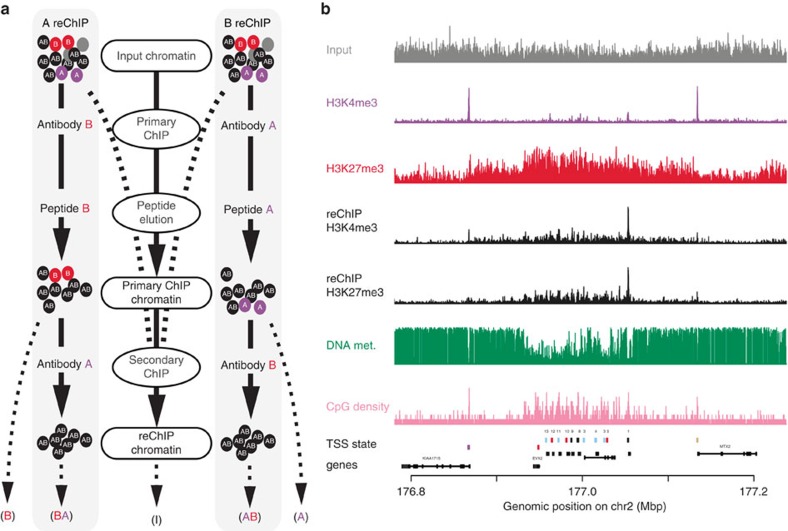
The reChIP-seq method. (**a**) Experimental design. Black, purple, red and grey circles denote chromatin containing A and B antigens, only A antigens, only B antigens, or neither A nor B antigens, respectively. (**b**) ChIP- and reChIP-seq at the human HOXD locus. The colours of the boxes in the TSS state track indicate the co-occupancy patterns as described in Fig. 2.

To alleviate this technical deficit, we have developed a reChIP-seq method that combines a conventional primary ChIP with a specific peptide elution. The eluted material is used in a secondary ChIP and the so-isolated DNA is sequenced (Fig. 1a, see ‘Methods' section). In brief, the efficiency and nucleosome specificity of this protocol relies on the following technical considerations: (i) the cross-linked input chromatin needs to be sheared to mono-/di-nucleosomal lengths to allow for an efficient elution (Supplementary Fig. 1); (ii) a defined number of cells should be used to estimate optimal antibody and peptide concentrations (Supplementary Fig. 2); (iii) the primary ChIPs are eluted with complementary peptides providing a specific and non-denatured template for the secondary ChIP; (iv) the paired-end sequencing libraries are size selected to enrich for DNA fragments of mono-nucleosomal length; and (v) bioinformatic analysis should be restricted to fragments longer than 120 and shorter than 240 base pairs.

Existing peak callers use the relatively uniformly distributed chromatin input as a control to delineate regions of enrichment achieved by conventional ChIP-seq. In reChIP-seq, the control corresponds to the primary ChIP with a more variable signal distribution. We developed an algorithm, referred to as normR (Supplementary Software, available also at https://bioconductor.org/packages/normr), which fits a binomial mixture model, where one component corresponds to the background and the other to the signal. In this way, normR normalizes reChIP-seq data on the basis of background regions to estimate enrichment and its statistical significance (see ‘Methods' section). It performs comparable to existing peak calling tools (Supplementary Tables 1-4) but identifies more enriched regions especially in (re)ChIP-seq experiments yielding a low signal-to-noise ratio (Supplementary Note 1, Supplementary Table 1). normR outperforms the other tested tools in identifying regions with high log_2_ (re)ChIP over Input fragment counts (Supplementary Figs 3 and 4). Furthermore, it offers the flexibility to define arbitrary genomic regions such as TSSs or CpG islands.

To illustrate the utility of the reChIP-seq and normR approach, we identified bivalently modified nucleosomes in TCMs. To this end, we performed ChIP-seq for H3K4me3 and H3K27me3, which served as control for H3K4me3 reChIP-seq from H3K27me3 primary ChIP and H3K27me3 reChIP-seq from H3K4me3 primary ChIP, respectively. In addition, H3K4me1, H3K9me3, H3K27ac and H3K36me3 ChIP-seqs, whole-genome bisulfite sequencing (WGBS) and RNA-seq were performed using the same sample.

As a first reference, we examined H3K4me3 and H3K27me3 reChIP-seq enrichment at a classical bivalent locus, HOXD (Fig. 1b). We found that the TSSs of the HOXD genes showed strong enrichment in H3K4me3 and H3K27me3 ChIPs and reChIPs which was accompanied by hypomethylated CpG-rich DNA, indicating bivalency. These findings validate the ability of reChIP-seq and normR to detect and map combinatorial bivalent histone modifications at the mono-nucleosomal level.

### reChIP-seq and normR reveal bivalent TSSs in TCMs

The identification of genomic targets where two antigens co-occur is ideally based on two reciprocal reChIP-seq experiments, where antigen A is precipitated first followed by the precipitation of antigen B and vice versa. However, partial co-occupancy may also occur, when only one of the two reciprocal re-ChIPs shows enrichment over its primary ChIP^16^. This arises when only a minor fraction of A containing chromatin also contains B (denoted as AB). In this case, a primary B-ChIP will efficiently recover antigen A, which can then be enriched by the secondary A-ChIP. However, when antigen A is precipitated first only very few copies will also harbour B, leading to statistically negligible enrichment by the secondary B-ChIP. In these cases, partial co-occupancy can still be detected as both reciprocal reChIPs will exhibit enrichment over a chromatin input control.

**Figure 2:**
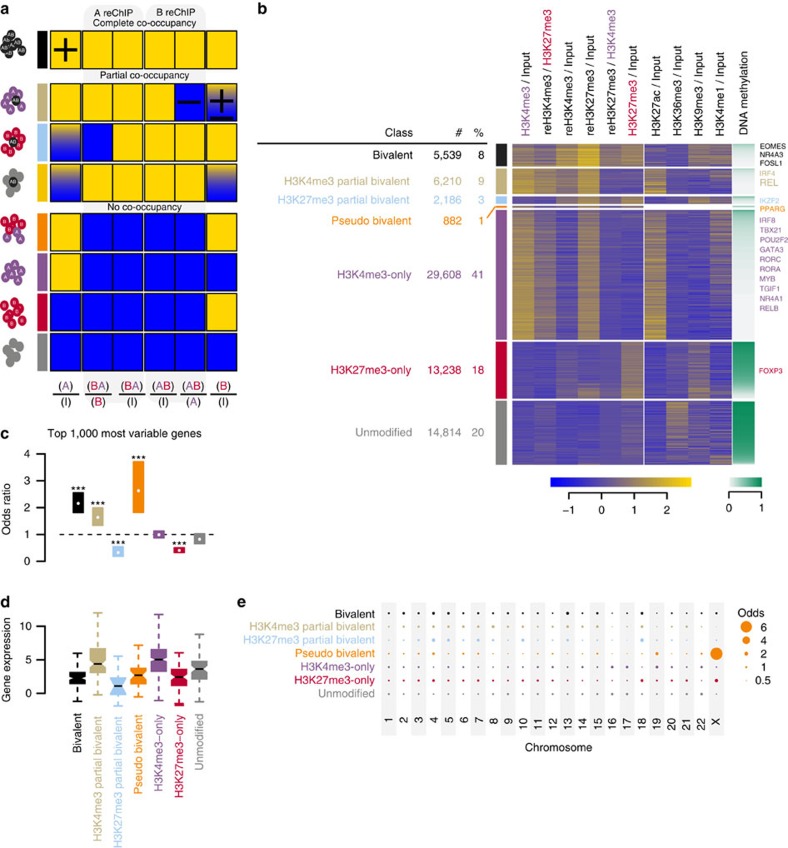
reChIP-seq and normR analysis reveals bivalent promoters in primary human CD4^+^ central memory T cells. (**a**) Anticipated outcomes after analysis. (X)/(Y), where X denotes (re)ChIP-seq track (A ChIP, B ChIP, BA reChIP or AB reChIP) normalized by the control Y (I=Input, A or B). Yellow, blue and blue/yellow boxes indicate enrichment, no enrichment or borderline enrichment, respectively. The colours next to the (re)ChIP enrichment patterns denote: full co-occupancy (black); A antigen partial co-occupancy (beige); B antigen partial co-occupancy (blue); low co-occupancy (yellow); pseudo co-occupancy (orange); only A antigen (purple); only B antigen (red); or no occupancy (grey). (**b**) TSS clustered by their enrichment pattern for H3K4me3, H3K27me3, reChIP H3K4me3 and reChIP H3K27me3 over respective controls resulting in seven classes (see **a**): black, full bivalent; beige, H3K4me3 partial bivalent; blue, H3K27me3 partial bivalent; orange, pseudo bivalent; purple, H3K4me3-only; red, H3K27me3-only and grey; unmodified. Within a class, the TSS are ordered from low (bottom) to high DNA methylation. The gene names on the right denote the gene state of critical regulators of T Helper subtype differentiation^45^. Enrichment was calculated by normR (see the ‘Methods' section) (**c**) Enrichment/Depletion analysis of the classes in the top 1,000 most variably expressed genes. Stars indicate statistical significance (***) P value <0.0001 (Two-sided Fisher's exact test). (**d**) Boxplots of gene expression values of the top 1,000 most variably expressed genes contingent on the class. (**e**) Enrichment/Depletion analysis of the classified TSSs on 23 Human chromosomes.

Considering all possible combinations, we anticipate eight different patterns for the normalized ratios of the primary ChIPs over input, reChIPs over their primary ChIPs and reChIPs over the input (Fig. 2a). These eight distinct patterns discriminate: (i) full co-occupancy leading to significant high signals in all six ratios (that is, primary ChIPs over input, reChIPs over their respective primary ChIP and reChIPs over input); (ii) partial co-occupancy, where either antigen A or B are predominantly enriched mixed with a smaller fraction of AB chromatin; or where loci are devoid of A and B, except for a small AB fraction. This later partial co-occupancy class was excluded from consideration, due to the uncertainty of whether it reflects low co-occupancy or a technical/computational artifact representing the false discovery rate of our approach (3 TSS out of 72,480; <1%); and (iii) no co-occupancy, where no enrichment is observed for both reChIPs and enrichment is observed in the primary ChIPs for A-only, B-only, A and B (pseudo co-occupancy) or no primary ChIP enrichment.

We examined H3K4me3, H3K27me3, reH3K4me3 and reH3K27me3 enrichments over controls at 73,043 TSS for protein-coding transcripts (GENCODE v19). After removing TSSs with no signal in the Input or in any of the eight (re)ChIP experiments, 72,480 TSSs remained. The counts in these TSSs served as input for normR to call enrichment of the (re)ChIPs over the respective controls. We performed all the analyses using *q* value thresholds of 0.001, 0.01 and 0.1, corresponding to 0.1%, 1% and 10% false discovery rate (FDR). Over this wide range of *q* value thresholds, the class assignments remain largely stable (Supplementary Fig. 5). Here, we show the results at a FDR of 1%, all results can be recapitulated at a FDR of 0.1% (Supplementary Fig. 6) and 10% (Supplementary Fig. 7). On grouping TSSs by their modification pattern outlined above, we obtained eight classes, where we discarded the low co-occupancy class as it contained only 3 out of the 72,480 TSSs (Fig. 2b and Supplementary Data 1).

The first class (black; *N*=5,539 or 8%) shows enrichment in all six ratios indicating full co-occupancy and thus is referred to as bivalent. The second class (beige; *N*=6,210 or 9%) shows enrichment in the H3K4me3 ChIP, both reChIPs over input and the H3K4me3 reChIP over the H3K27me3 primary ChIP but no enrichment for H3K27me3 over input or the H3K27me3 reChIP over the H3K4me3 primary ChIP. These TSSs are also enriched for H3K27ac, indicating transcriptional activity. The third class (blue; *N*=2,186 or 3%) shows an inverse pattern to the second class and the absence of H3K27ac indicating that they are transcriptionally silent. We refer to these classes as H3K4me3 and H3K27me3 partial bivalent, respectively. The fourth class (orange; *N*=882 or 1%) shows enrichment in both primary ChIPs, but no enrichment in the reChIPs, indicating no co-occupancy and is denoted as pseudo bivalent. Note that by co-enrichment, class 4 TSSs would have been called bivalent resulting in a false positive rate of ∼14%. The fifth class (purple; *N*=29,608 or 41%) shows H3K4me3 and H3K27ac enrichment indicating that they are active. The sixth class (red; *N*=13,238 or 18%) only shows H3K27me3 enrichment indicating that they are inactive. The majority of these TSSs are DNA hypermethylated, except a small subclass with low DNA methylation that correspond to high CpG promoters (Supplementary Fig. 8). Interestingly, this small divergent subclass shows a similar pattern to the H3K27me3 partial bivalent TSSs, indicating that they are at the border between the H3K27me3-only and the H3K27me3 partial bivalent class. Finally, the seventh class (grey; *N*=14,814 or 20%) shows no enrichment in the (re)ChIPs and is referred to as unmodified. This class, however, shows H3K36me3 enrichment, a mark that is associated with transcriptional elongation^27^ indicating that many of these TSSs are internal to an upstream active TSS.

The two partial bivalent and the pseudo bivalent patterns may reflect heterogeneity in the TCM population. The abundance and epigenomic state of these subpopulations depend on the immunological history of both single cell clones as well as of the donors and, therefore, could be heterogeneous within the total cell population. We analysed the gene expression variance in an independent sample of CD4^+^ TCMs from five donors^28^. We calculated the coefficient of variation to obtain a normalized measure of variability, extracted the top 1,000 most variable genes and determined the number of genes falling into each of the seven classes (see the ‘Methods' section).

Bivalent, H3K4me3-partial bivalent and pseudo bivalent genes are over-represented in these genes, H3K27me3 partial bivalent and H3K27me3-only genes are depleted and H3K4me3-only and unmodified are as frequent as expected by chance (Fig. 2c). This assignment remains largely unaffected by different choices of the top *K* most variable genes (Supplementary Fig. 9). The high variability in H3K4me3 partial and pseudo bivalent genes is consistent with the idea that these arise due to heterogeneity on the cellular or allelic level. The low variability of H3K27me3-only and H3K27me3 partial bivalent genes is also expected as they are stably repressed by Polycomb and heterogeneity in the latter is between two repressed states. However, the high variability in bivalent genes is unexpected. To investigate the reason for this, we analysed the gene expression levels for these top 1,000 genes contingent on the promoter class. The bivalent genes are lower expressed than H3K4me3 partial and pseudo bivalent genes (Fig. 2d) indicating that the high gene expression variation in the bivalent genes may be due to gene expression noise rather than sample heterogeneity, whereas H3K4me3 partial bivalent and pseudo bivalent gene expression variation can be explained by subpopulation heterogeneity.

Another source of heterogeneity may be allele-specific expression. Consistently, pseudo bivalent TSSs accumulate on the X chromosome (Fig. 2e). Given that the TCMs have been isolated from female donors, H3K4me3 and H3K27me3 are likely present on the active and inactive X, respectively.

These findings indicate that a surprising large number (∼20%) of the 72,480 human TSSs are either partial or full bivalent in TCMs. Furthermore, while the statistical inference of co-occupancy by intersection of independent ChIPs is astonishingly specific (false positive rate ∼14%), it is not very sensitive, as it missed ∼60% of co-occupied regions (partial bivalent regions), due to the fact that either H3K4me3 or H3K27me3 ChIP showed no statistical significant enrichment. Only the reChIP approach achieved a sufficient sensitivity in detecting these signatures, which indicates that technical and/or statistical problems may preclude the detection of bivalency from the intersection of conventional ChIPs.

### Characterization of genes driven by bivalent promoters

**Figure 3:**
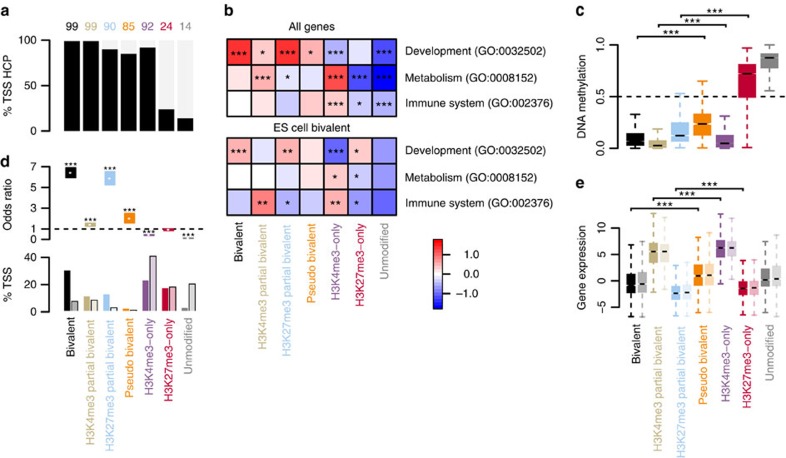
Functional characterization of genes driven by bivalent promoters. (**a**) Distribution of the seven TSS classes in High CpG content promoters (HCPs). (**b**) GO-term enrichment of the seven classes for GO:0032502 (developmental process), GO:0008152 (metabolic process) and GO:0002376 (immune system process). Shown are the calculated log_2_ fold enrichment in the respective terms indicated by the colour (see colour key). Top, for all genes; bottom for genes annotated to be bivalent by BGDB^29^. Stars indicate statistical significance: (***) P value <0.0001, (**) P value <0.001 and (*) P value <0.05 (Two-sided Fisher's exact test). (**c**) Boxplots of DNA methylation levels contingent on the seven promoter classes. (***) P value <2.2e−16 (Wilcoxon signed-rank test). (**d**) Top, enrichment/depletion analysis of BGDB bivalent genes in the seven classes. Stars indicate statistical significance as in **b**; bottom, percentage of TSS in the different classes. Dark bars denote TSS overlapping with the BGDB bivalent genes; fair bars denote the genomic distribution. (**e**) Boxplots of gene expression for the different classes. Dark coloured, gene expression measured in the sample used for (re)ChIP; fair coloured, gene expression average over five independent central memory T-cell samples^28^.

Bivalency in ESCs predominantly marks so-called high CpG content promoters (HCPs) of developmental regulators^6^. Similarly, in TCMs, almost all bivalent and H3K4me3 partial bivalent and the vast majority of H3K27me3 partial bivalent TSSs are HCPs (Fig. 3a), and are associated with developmental functions (Fig. 3b, top). H3K4me3 partial bivalent genes in TCMs are also associated with metabolic functions as are H3K4me3-only genes, which are additionally associated with immune system functions. Thus, in TCMs bivalency targets are developmental regulators as in ESCs but do not preferentially involve regulators of the different T helper cell subtypes (Supplementary Table 5).

Furthermore, bivalent CpG-rich domains are known to be hypomethylated in ESCs. We examined DNA methylation levels across the seven TSS classes to check whether this holds true in TCMs (Fig. 3c). We found that unmodified and H3K27me3-only TSSs were highly DNA methylated, whereas H3K4me3-only and all bivalent states are DNA hypomethylated. Furthermore, full bivalent TSSs were significantly less DNA methylated than pseudo bivalent. H3K4me3 partial bivalent TSSs yielded significantly lower DNA methylation levels than H3K4me3-only ones (Fig. 3c and Supplementary Fig. 10)—an observation that is also true for H3K27me3 partial bivalent compared with H3K27me3-only. Thus, the bivalent state coincides with DNA hypomethylation at TSSs in TCMs as in ESCs.

These results suggest a functional overlap between bivalent genes in TCMs and ESCs and argue against a TCM-specific function of bivalency. To test this idea, we examined the overlap between bivalent genes in human ESCs as identified by the Bivalent Gene Database (BGDB)^29^ with the TSS states in TCMs. This comparison revealed that >50% of the bivalent ESC genes are bivalent (30%), H3K4me3 partial (11%) or H3K27me3 partial bivalent genes (13%) in TCMs, that is, ESC bivalent genes are over-represented in these three TCM bivalent classes (Fig. 3d). Most of the remaining ESC bivalent genes resolve either to H3K27me3-only (17%) or H3K4me3-only (23%) and only 3% become unmodified in TCMs. This shows that most bivalent TSSs in ESCs remain at least partially bivalent in TCMs and that ∼40% resolve to either an H3K4me3- or H3K27me3-only state—a phenomenon that has been observed during ESC differentiation^6^. It is interesting to note that TCMs harbour many more partial and full bivalent TSSs (between 77 and 93% are TCM-specific), indicating either that they were not detected before or that the bivalent state was gained during development. The latter is consistent with observations of Mohn *et al*. that bivalency can be gained during development^7^.

Further evaluation of the associated functional terms revealed that the genes that remain bivalent, become H3K27me3 partial bivalent or resolve to H3K27me3-only are associated with developmental functions. The genes that resolve to H3K4me3-only are depleted in developmental functions (Fig. 3b, bottom), but interestingly are, like H3K4me3 partial bivalent genes, associated with immune system functions. These findings demonstrate that bivalency in both ESCs and TCMs targets developmental genes, whereas those that resolve into the H3K4me3-only state drive cell-type-specific genes.

Bivalency in ESCs is affiliated with transcriptional repression. We therefore wanted to explore the effect of bivalency on transcription in TCMs (see the ‘Methods' section). We found that H3K4me3-only and H3K4me3 partial bivalent genes are transcriptionally active (Fig. 3e) consistent with H3K27ac enrichment in these classes (Fig. 2b). In contrast, full bivalent, H3K27me3 partial bivalent, H3K27me3-only and unmodified genes are associated with no or little transcriptional activity. Bivalent genes showed a significantly lower gene expression level than pseudo bivalent genes supporting the idea that the latter are active (H3K4me3-only) in some cells or alleles and repressed (H3K27me3-only) in others. A further comparison of H3K4me3-only with H3K4me3 partial bivalent and H3K27me3-only with H3K27me3 partial bivalent genes revealed that the expression levels of partial bivalent TSSs are significantly lower. These results are confirmed by an independent RNA-seq study measuring gene expression in CD4^+^ TCMs of five donors^28^ (Fig. 3e). Thus, similar to ESCs, bivalency in TCMs leads to transcriptional repression.

Together these findings indicate that characteristic features of the bivalent state in human ESCs, namely transcriptional repression, CpG-richness, DNA hypomethylation and association with the TSSs of developmental regulators, are largely maintained in TCMs. Furthermore, they demonstrate that the full, H3K4me3 partial and H3K27me3 partial bivalent classes show distinct characteristics, which are different from the pseudo bivalent, H3K4me3-only and H3K27me3-only classes underlining the added benefit of reChIP-seq and our analysis.

### Sequence characteristics of bivalent promoters

**Figure 4:**
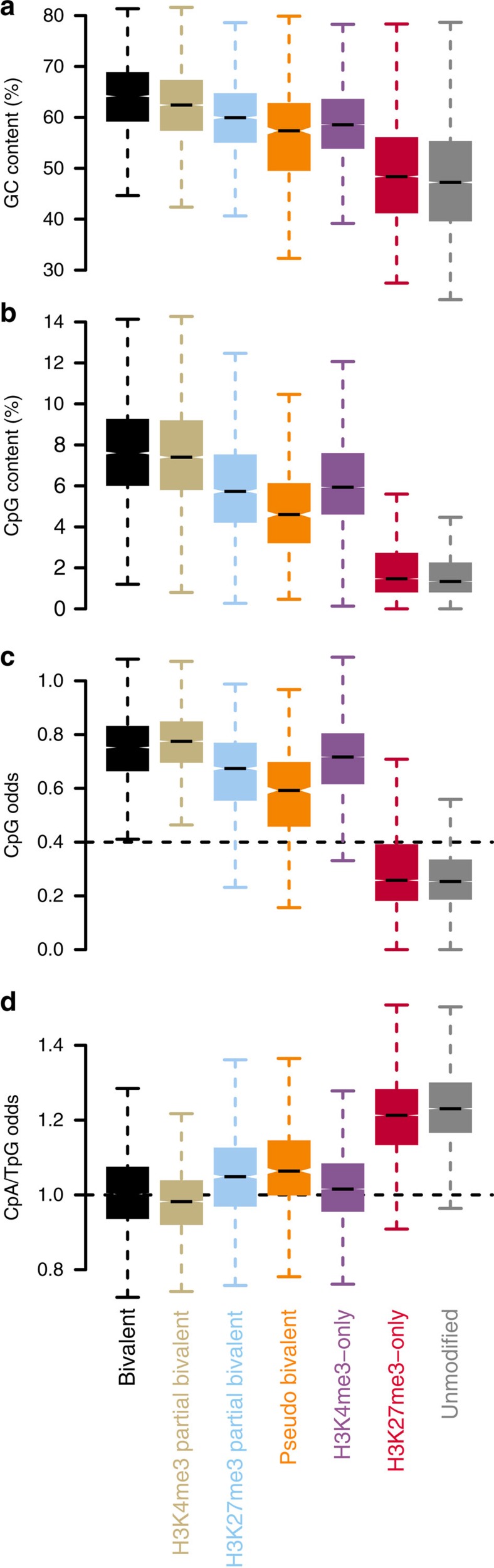
Sequence characteristics of bivalent promoters. (**a**) Boxplots of the G+C content in percentage, (**b**) the CpG content in percentage, (**c**) the CpG odds, that is, observed CpG count over the expected CpG count and (**d**) the CpA/TpG (deamination products of methylated CpGs) odds.

The conservation of the bivalent state between ESCs and TCMs at hypomethylated HCPs suggests that it may be determined by the DNA sequence. A comparison of G+C and CpG content stratified by the seven promoter classes revealed that full and partial bivalency is associated with a high G+C and CpG content, while unmodified and H3K27me3-only coincides with a low G+C and CpG content (Fig. 4a,b). To account for the high correlation between G+C and CpG content (Supplementary Fig. 11), we calculated the odds ratio of observed CpG content over expected CpG content from the G+C content. The statistical association of bivalency with CpG remains after accounting for the effect of the G+C content (Fig. 4c), indicating that a high G+C content and a high CpG content is characteristic of bivalency.

Next, we turned to CpA/TpG odds, which are negatively correlated to the CpG odds (Supplementary Fig. 12). CpA and TpG are the products of deamination of methylated CpGs and their over-representation indicates germline DNA methylation and deamination. H3K4me3-only, full bivalent and partial bivalent promoters show a median odds ratio of ∼1 (Fig. 4d), indicating no over-representation of CpA/TpG in these TCM TSS classes and arguing that their DNA hypomethylated state is present in the germline. In contrast, CpA/TpG are over-represented in unmodified and H3K27me3-only promoters (median odds ratio >1.2), indicating germline DNA methylation and deamination. Together, these findings support that DNA hypomethylation, high C+G and high CpG content is conserved in the germline and defines essential prerequisites for the establishment of bivalency in ESCs and TCMs.

### Bivalency is the response to unmethylated CpG-rich DNAs

**Figure 5:**
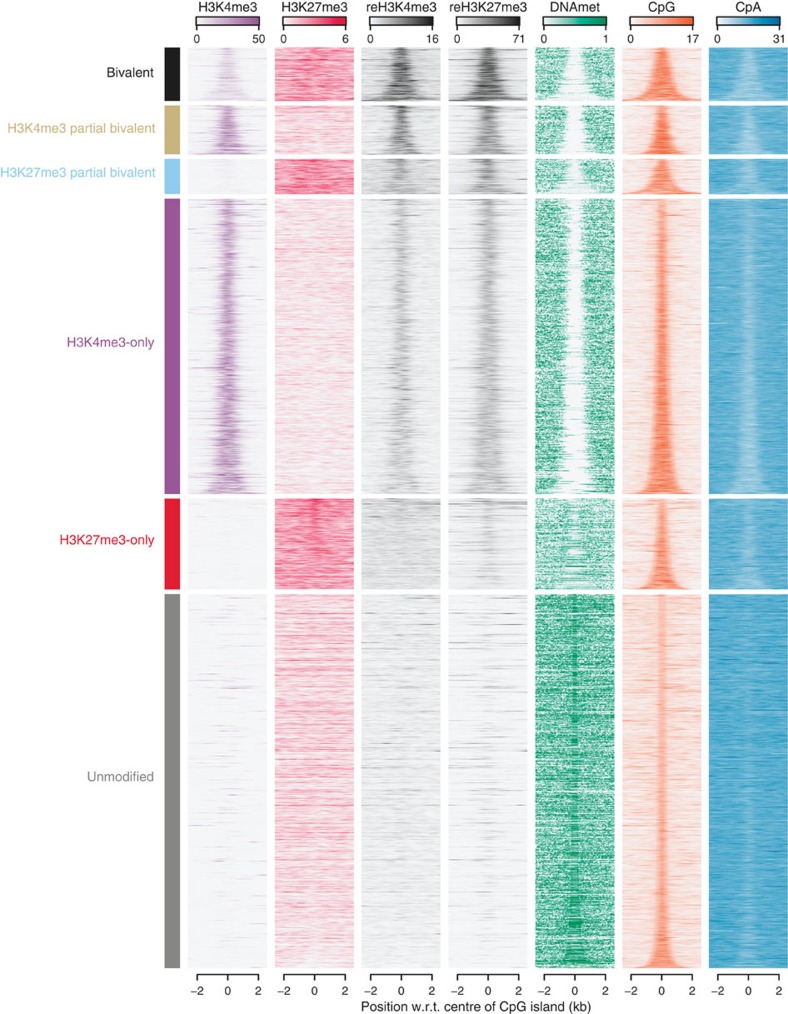
Bivalency is the default response to unmethylated CpG-rich DNA sequences. CpG islands clustered by their enrichment pattern for H3K4me3, H3K27me3, reChIP H3K4me3 and reChIP H3K27me3 over respective controls. Fragment coverage for the tracks indicated above each column (1 to 4) for each CpG island (rows, sorted by CpG island length) ±2,000 base pairs centred around the centre of the CpG island. The fifth column represents DNA methylation (0 denotes no DNA methylation and 1 denotes full DNA methylation). The last two columns represent the CpG content and the CpGA/TpG content in percentage.

To disentangle the epigenetic and genetic requirements for bivalency, we classified 27,537 CpG islands in the human genome defined by their sequence characteristics^30^ into the aforementioned enrichment patterns for H3K4me3, H3K27me3, reH3K4me3 and reH3K27me3 (Supplementary Data 2). Different *q* value thresholds led to limited changes in the classification (Supplementary Fig. 13). We performed the analysis using *q* value thresholds *q*<0.001 (Supplementary Fig. 14), *q*<0.01 (Fig. 5) and *q*<0.1 (Supplementary Fig. 15). As only 115 CpG islands were classified to low co-occupancy and 115 to be pseudo bivalent, we removed these two classes from further analysis. Bivalent CpG islands strongly overlapped with the reChIPs and H3K4me3 (full bivalent: *N*=1,681 or 6% of CpG islands, H3K4me3 partial bivalent: *N*=1,540 or 6% and H3K27me3 partial bivalent: *N*=1,149 or 4%). These showed either no or low levels of DNA methylation. Similarly, ∼32% of the CpG islands were H3K4me3-only (*N*=8,672), which likewise showed either no or low levels of DNA methylation. The remaining 51% of CpG islands were H3K27me3-only (*N*=2,757 or 10%) or unmodified (*N*=11,508 or 41%) and showed high DNA methylation levels. These patterns demonstrate that hypomethylated CpG islands coincide with H3K4me3 and the bivalent state genome-wide even when decoupled from promoters. The conservation of these features together with the fact that bivalent and H3K4me3-only CpG islands in TCMs show a depletion of CpA/TpG di-nucleotides (Fig. 5) strongly argues for their germline DNA hypomethylation and indicates a stabilizing interplay between the genome and the epigenome.

## Discussion

**Figure 6:**
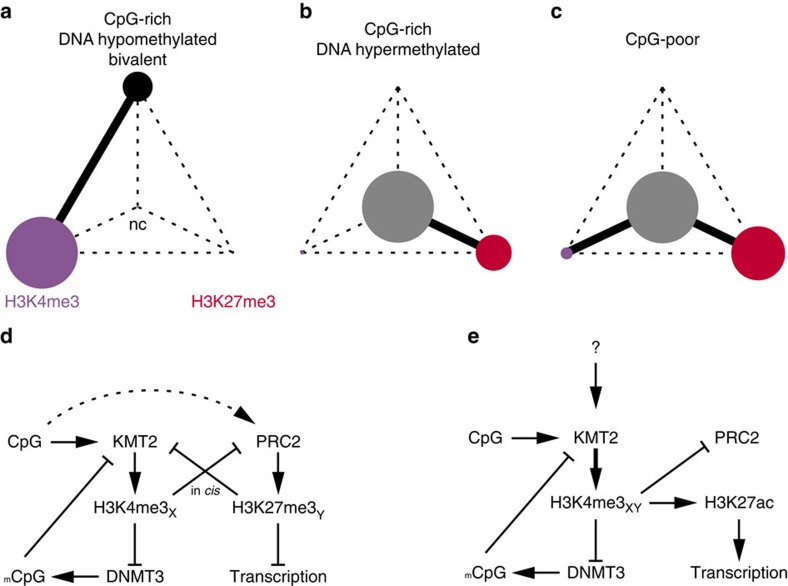
Cross-talk between genome and epigenome. (**a**–**c**) The filled circles denote the relative fraction of TSSs in the indicated state. Purple indicates H3K4me3-only, red H3K27me3-only, black bivalent and grey the unmodified state. Continuous lines indicate possible transitions, while broken lines indicate a low probability of a transition between the states. (**d**,**e**) Possible models promoting either a bivalent (**d**) or H3K4me3-only state (**e**) as depicted in **a**. H3K4me3_X_ and H3K27me3_Y_ indicate that they reside on two different H3-tails, while H3K4me3_XY_ indicates that H3K4me3 is on both H3-tails of a nucleosome.

We have developed and applied a reChIP-seq protocol and the normR algorithm to analyse and dissect modes of H3K4me3 and H3K27me3 bivalency at the nucleosomal level on a genome-wide scale. Using this method, we could unambiguously assign various types of bivalency occurring at developmental promoters in TCMs. Similar to ESCs, we found that hypomethylated CpG-rich promoters are either H3K4me3-only and transcriptionally active or bivalent and transcriptionally repressed. This dual mode indicates that hypomethylated CpG-rich promoters can switch between a bivalent off and a H3K4me3 on state (Fig. 6a). On the contrary, DNA hypermethylated CpG-rich promoters/regions are either unmodified or show an H3K27me3-only state (Fig. 6b). Likewise, CpG-poor promoters are predominantly unmodified or H3K27me3-only, with only a minor fraction carrying H3K4me3 (Fig. 6c).

On the basis of our findings as well as the work of others, we conclude that the bivalent state is a dual repression control mechanism, where H3K27me3 represses transcription and H3K4me3 suppresses DNA methylation (Fig. 6d,e). Unmethylated CpGs recruit H3K4me3 methyltransferases^31^ and H3K4me3 inhibits DNA methylation by preventing the recruitment of *de novo* DNA methyltransferases^32^,^33^,^34^, which creates a feedback loop stabilizing the DNA hypomethylated state. In the absence of activating transcription factors^35^, these CpG-rich sequences gain H3K27me3 leading to the bivalent state and transcriptional repression (Fig. 6d); whether the acquisition of H3K27me3 depends on H3K4me3 or not remains unclear. This state is not static and the binding of transcriptional activators at these regions promotes H3K27me3 removal and its replacement by H3K27ac leading to transcriptional activation (Fig. 6e). The DNA hypomethylated state of H3K4me3-only and bivalent classes appears to be conserved in the germline, leading us to hypothesize that the feedback cycle between DNA hypomethylation and H3K4me3/bivalency is a mechanism to protect against excessive mutations resulting from deamination in developmental promoters. Collectively, these mechanisms thereby reflect a robust interplay between genetic and epigenetic inheritance.

The prerequisite for our analysis was the development of a reChIP-seq protocol to discern the co-localization of two proteins at a single-nucleosome resolution and a tailored bioinformatic analysis. The combination of both facilitates the separation of true co-localizations from those cases where the co-enrichment of the two antigens is not due to physical co-localization. Moreover, it provides a very sensitive tool to discern heterogeneous co-occupancy, which remained undetected by the co-enrichment of primary ChIPs. The reChIP-seq approach requires peptide-derived antibodies or monoclonal antibodies with known epitope, which may limit its applicability. Peptide elution yields non-denaturated native chromatin for the secondary ChIP in contrast to previous procedures that relied on elution by, for example, dithiothreitol^14^,^17^, which denatures the chromatin. As a result, our approach alleviates the requirement of large amounts of input chromatin^18^ but may still not be efficient enough for low abundance chromatin features. Thus, the proposed reChIP-seq approach has a broad application range, especially when sample amounts are limited, but requires antibodies with defined epitopes and the corresponding peptide to efficiently elute the captured chromatin.

A specific elution of enriched chromatin from primary ChIPs by peptide competition is the principal technical requirement for an efficient reChIP. The elution, however, becomes increasingly difficult when multiple antigens are present on the precipitated chromatin template resulting in many interactions that have to be broken simultaneously. This problem can be diverted by shearing the chromatin to mono/di-nucleosomal levels, thereby limiting the antigen–antibody interactions. This reduction becomes particularly important when analysing broad histone marks such as H3K27me3, where a series of neighbouring nucleosomes contain the primary target antigen. However, when this caveat is taken into consideration and the chromatin is sheared appropriately, our reChIP approach functions as demonstrated here for H3K27me3.

Overall, our results demonstrate that reChIP-seq and the proposed analysis have a great potential to provide detailed biological insight into the co-localization of proteins and/or their modifications and may be instrumental to decode the combinatorial complexity in histone modification patterns and their functional consequences.

## Methods

### Isolation of primary human central memory T cells

Buffy coats from female donors were obtained from the DRK Blutspendedienst, Berlin, Germany. Use of buffy coats for scientific purposes in the DEEP project was approved by the ethics committee of the Charite Universitaetsmedizin Berlin (Ethikkommission, Ethikausschuss 1 am Campus Charite Mitte) under the application number EA1/095/13. Informed consent was given by the donors. Peripheral blood mononuclear cells were separated using a LSM 1077 density gradient (PAA, GE Healthcare). Remaining erythrocytes were lysed using the Buffer EL (Qiagen). CD4^+^ T cells were pre-enriched using anti-CD4 microbeads and the AutoMACS magnetic separation system (Miltenyi Biotec). MACS-sorted CD4^+^ T cells were surface stained using the following directly conjugated antibodies (all purchased from BioLegend, except CD45RA that was purchased from Beckman Coulter): CD3 (clone: UCHT1), CD4 (OKT4), CD25 (BC96), CD45RA (2H4LDH11LDB9), CD62L (DREG-56), CD127 (A019D5). The CD4^+^ central memory T cells were sorted as outlined in Supplementary Fig. 16a according to the surface marker combination CD3^+^CD4^+^CD25^low^CD45RA^−^CD62L^+^ by FACS (Aria, BD-Bioscience). Purity of the sorted populations was confirmed by flow cytometry (Supplementary Fig. 16a). TCMs from three donors were pooled and then used to perform the bivalency ChIP, RNA-seq and whole-genome bisulfite sequencing experiments.

### The reChIP-seq procedure

The cells were crosslinked in 1% formaldehyde at a concentration of 1 × 10^6^ cells per ml for 5 min at room temperature under rotation, followed by quenching in 0.125 M glycine for 10 min. The crosslinked cells were then pelleted by centrifugation at 16.9*g* for 5 min at 4 °C.

The reChIPs were performed with 4 × 10^6^ primary CD4^+^ central memory T cells per primary ChIP. A total of 4 × 10^6^ cells were lysed in 133 μl 1% SDS lysis buffer (100 mM NaCl, 50 mM Tris-HCl pH 8.1, 5 mM EDTA, 0.2% NaN_3_, 1% SDS and 3% Triton X) for 10 min on ice. The cell lysate was then diluted to 0.33% SDS (or 400 μl) with ChIP Dilution buffer (50 mM Tris-HCl pH 8.6, 100 mM NaCl, 5 mM EDTA and 0.2% NaN_3_) and resuspended several times using a syringe. The cell lysate was then divided into 200 μl aliquots, transferred to 1.5 ml TPX tubes (Diagenode C30010010-1000) and sheared on a Bioruptor Plus at high intensity for 3 × 9 cycles. The sheared chromatin lysate was then centrifuged at 16.9*g* for 20 min at 4 °C to pellet debris.

Four hundred microlitres of chromatin (4 × 10^6^ cells) was diluted with ChIP dilution buffer to 1,200 μl (0.08% SDS) before the addition of the antibody bead complexes. Four micrograms of polyclonal antibodies H3K4me3 (Diagenode: C15410003-50), H3K27Ac (Diagenode: C15410196) or H3K27me3 (Diagenode: C15410195) was incubated with 20 μl of DiaMag Protein A coated magnetic beads (Diagenode C03010020-150) for 1 h at 4 °C under rotation and then washed several times with ChIP dilution buffer to remove unbound antibodies before the addition of chromatin. The chromatin lysate (400 μl) and antibody bead complexes were then incubated for 24 h under rotation at 4 °C. The supernatant was then collected and re-incubated for 24 h at 4 °C under rotation with fresh antibody bead complexes to ensure maximal epitope depletion from the chromatin lysate. The beads containing the precipitated chromatin were then washed extensively in ChIP wash buffers 1–4 (ChIP wash buffer 1: 0.1% SDS, 1% Triton X-100, 2 mM EDTA, 20 mM Tris-HCl pH 7.5 and 150 mM NaCl, ChIP wash buffer 2: 0.1% SDS, 1% Triton X-100, 2 mM EDTA, 20 mM Tris-HCl pH7.5 and 500 mM NaCl, ChIP wash buffer 3: 0.25 M LiCl, 1% NP40, 1% Na deoxycholate, 1 mM EDTA and 10 mM Tris-HCl pH 7.5 and ChIP wash buffer 4: 50 mM Tris-HCl pH 7.5 and 10 mM EDTA). The beads were then transferred to a new Eppendorf tubes and H3K4me3 and H3K27me3 containing chromatin was eluted from the beads with either 2 μg of H3K4me3 peptide (Abcam ab1342) or H3K27me3 peptide (Abcam ab1782), respectively, in 400 μl of ChIP dilution buffer, supplemented with 0.1% SDS overnight at 4 °C under rotation. A total 20% of the eluted chromatin was then retained as the primary ChIP. The remaining 80% of the eluted material was then used for the reChIPs and was added to antibody bead complexes containing 1μg of H3K4me3 (Diagenode: C15410003-50), H3K27ac (Diagenode: C15410196) or H3K27me3 (Diagenode: C15410195) and 20 μl of DiaMag Protein A coated magnetic beads (Diagenode C03010020-150). The H3K4me3 eluted chromatin was reChIPed for H3K27me3 and the H3K27me3 eluted chromatin was reChIPed for H3K4me3, for 24 h under rotation at 4 °C. The beads containing the precipitated bivalent chromatin were then washed extensively in ChIP wash buffers 1–4, transferred to new Eppendorf tubes and de-crosslinked in 100 μl TE buffer pH 9.5 (50 mM Tris-HCl pH 9.5 and 10 mM EDTA) at 65 °C overnight, followed by 1 h of RNAseA treatment (10 ng μl) at 55 °C and 3 h Proteinase K treatment (3 μl) at 55 °C. The DNA was then purified from the solution via phenol chloroform precipitation overnight, lyophilized by a speed vacuum and resuspended in 11 μl of nuclease free dH20. The purified DNA was quantified by Quibit and ChIP libraries were generated using Diagenode MicroPlex library preparation kits (C05010010) according to the manufacturer's instructions. The libraries were paired-end sequenced on an Illumina HiSeq 2500 platform.

### ChIP qPCR primer sequences

P53: Forward: 5′-GGGTGGATGTGCAAAGAAGT-3′

Reverse: 5′-GGGTGTAGATGATGGGGATG-3′

P53Int: Forward: 5′-CTGTGGGTTTGGTGTGTGAG-3′

Reverse: 5′-GTTGCAGGTTACAGGGCAAT-3′

Rad51: Forward: 5′-TCTTCTCGAGCTTCCTCAGC-3′

Reverse: 5′-AGCGCTCTTGTGGTTTGTTT-3′

Sox17: Forward: 5′-TTTCATGGTGTGGGCTAAGG-3′

Reverse: 5′-ACCCAGCATCTTGCTCAACT-3′

Ovol1: Forward: 5′-CACAGAGCTGTGGTCTCTGG-3′

Reverse: 5′-AGATTGGGAAGACGTGGATG-3′

Wnt4: Forward: 5′-GAGTCGTTTGCTCTCGGAAC-3′

Reverse: 5′-TGGAAGGAGTGTGTTCGTTG-3′

P16: Forward: 5′-CGACTTCAGGGGTGCCACATTC-3′

Reverse: 5′-TCTTTCCAGGCAAGGGGACGC-3′

### RNA-seq

Starting from 2 × 500 ng totalRNA of RIN 9.6, one-stranded totalRNA and one-stranded mRNA library were prepared according to the manufacturer's instructions (Illumina, San Diego, CA, USA). In brief, ribosomal RNA was depleted using the RiboZero Gold probes (for totalRNA library) and polyadenylated RNA was enriched using oligo-dT beads (for mRNA library). After chemical fragmentation of both libraries, first- and second-strand cDNA synthesis was performed, followed by mono-adenylation of 3′ ends and adapter ligation. The final library was PCR amplified and QC'ed on a Bioanalyzer. Both the libraries were sequenced for 2 × 101 nt on an Illumina HiSeq 2000, yielding about 100 million paired-end reads for each library.

### Whole-genome bisulfite sequencing

To achieve even sequencing coverage across the genome, two types of sequencing libraries were prepared for WGBS. For the first type of WGBS library, 100 ng of genomic DNA were used with the TruSeq methylation kit (Illumina) according to the manufacturer's protocol.

For the second type, we used the protocol from the TruSeq DNA PCR-Free Library Preparation Kit (Illumina) with some modifications: In a first step, 2 μg of DNA were sheared to fragment sizes of 300–500 bp using a Bioruptor NGS device (Diagenode, Liège, Belgium). Sheared fragments were purified with Ampure beads XP (Beckman Coulter, Brea, CA, USA) using a two-step procedure and then subjected to end-repair, A-tailing and NGS-adaptor ligation as described in the manufaturer's protocol. Ligations were bisulfite converted using EZ DNA methylation Gold kit (Zymo, Irvine, CA, USA) and then PCR amplified in six independent reactions, each consisting of 7 μl of sample, 5 μl of 10x Hot Star Taq buffer (Qiagen, Hilden, Germany), 5 μl of dNTPs (2.5 mM each; Thermo Fisher Scientific, Waltham, MA, USA), 1 μl of 10 μM forward primer (5′-AATGATACGGCGACCACCGAGATCTACAC-3′; Metabion, Planegg, Germany), 1 μl of 10 μM reverse primer (5′-CAAGCAGAAGACGGCATACGAGAT-3′; Metabion), 0.5 μl of Hot Star Taq (Qiagen) and 30.5 μl of aqua bidest. After an initial activation step (15 min at 95 °C), the reactions were amplified for nine cycles (30 s at 95 °C, 30 s at 60 °C and 1 min at 72 °C), followed by a final elongation step (7 min at 72 °C) and finally hold at 4 °C. The reactions were pooled and then purified with Ampure beads. Libraries were checked on a Bioanalyzer (Agilent Technologies, Santa Clara, CA, USA).

The first library type was loaded on one lane, the second library type on two lanes of a paired-end V3 flowcell (Illumina). Sequencing was performed on a HiSeq2500 generating 2 × 100 bp paired-end reads. In total, 1,610,652,952 reads were produced (∼40.1 × coverage) of which 1,508,942,939 (93.67%) were mapped using BWA^36^. For the bisulfite conversion efficiency, we calculated 99.92% using all HCH-positions and methylation calls were generated with Bis-SNP^37^.

### Mapping of (re)ChIP-seq paired-end reads

Paired-end reads from sequencing chromatin Input, primary ChIP and reChIP libraries were mapped to the human genome version hs37d5 with cnybwa version 0.6.2, which is a hardware re-implementation of bwa^36^ version 0.6.2. Mapping was separately performed for the mates on convey machines:

cnybwa-0.6.2 aln –t 12 –q 20 hs37d5-reference.fa \

sample_R1.fastq.gz > *sample*_R1.sai

cnybwa-0.6.2 aln –t 12 –q 20 hs37d5-reference.fa \

sample_R2.fastq.gz > *sample*_R2.sai 

where sample corresponds to the chromatin Input, primary ChIP and reChIP sample and R1 corresponds to the first and R2 to the second mate.

The resulting single-end *.sai files were paired:

bwa sampe -P -a 1000 -T -t 8 -r readgroupinformation \

hs37d5-reference.fa *sample*_R1.sai *sample*_R2.sai \

sample_R1.fastq.gz sample_R2.fastq.gz > sample_Sampe_output

allowing for a maximal insert size of 1,000 base pairs (−a 1,000). The readgroupinformation is initialized in the script as ‘@RG\tID:$ID\tSM:$SM\tLB:$LB\tPL:ILLUMINA', where $ID is composed of run and lane (for example, run130917_SN535_0184_B_D22FWACXX_51_Hf01_BlCM_Ct_reH3K27me3_ B_1_GCCAAT_L006), $SM the sampletype (for example, replicate1-reH3K27me3_51_Hf01_BlCM_Ct), and $LB the library (for example, sample_replicate1-reH3K27me3_51_Hf01_BlCM_Ct).

The resulting *sample*_Sampe_output was piped into samtools^38^ version 0.1.19 to convert SAM to BAM and sort by coordinate:

cat sample_Sampe_output | \

samtools view -uSbh - | \

samtools sort -o - > *sample*.bam

Duplicates were marked using Picard tools version 1.125:

java -Xmx50G -jar picard.jar MarkDuplicates I=*sample*.bam \

OUTPUT={*sample*.GALv1.date.bam} \

VALIDATION_STRINGENCY=SILENT \

REMOVE_DUPLICATES=FALSE \

ASSUME_SORTED=TRUE \

CREATE_INDEX=TRUE \

MAX_RECORDS_IN_RAM=12500000 \

METRICS_FILE={*sample*.PicardMarkDupmetrics.date.txt}

### Mapping of RNA-seq paired-end reads

The RNA-seq paired-end reads were mapped using tophat^39^ version 2.0.11:

tophat -p 8 --library-type fr-firststrand \

--b2-very-sensitive hs37d5.bowtie2-reference \

sample_R1.fastq.gz sample_R2.fastq.gz

We downloaded RNA-seq data for CD4^+^ central memory T cells using accession numbers ERS403433, ERS403431 ERS403284, ERS403403 and ERS403407 (ref. 28). We mapped the reads using STAR^40^ (version 2.5.1b_modified):

STAR --runmode alignReads \

--genomeDr hs37d5 \

--readFilesCommand zcat \

--outSAMtype BAM SortedByCoordinate \

--readFilesIn ERR_ID_1.fastq.gz ERR_ID_2.fastq.gz

### Whole-genome bisulfite sequencing (WGBS)

The WGBS paired-end reads were first trimmed by the default adaptor using SeqPrep version 0.4:

SeqPrep AGATCGGAAGAGCGGTTCAG \

-f {*sample*_R1.fastq.gz} \

-r {*sample*_R2.fastq.gz} \

-1 {*sample*.trimmed.R1.fastq} \

-2 {*sample*.trimmed.R2.fastq}

The trimmed reads were *in silico* bisulfite converted using methylCtools version 0.9.2:

methylCtools fqconv *sample*.trimmed.R1.fastq \

*sample*_R1.conv.fastq

methylCtools fqconv *sample*.trimmed.R2.fastq \

sample_R2.conv.fastq

These served as input to cnybwa version 0.6.2

cnybwa-0.6.2 aln –t 12 –q 20 hs37d5_PhiX_Lambda.fa \

sample_R1.conv.fastq > *sample*_R1.sai

cnybwa-0.6.2 aln –t 12 –q 20 hs37d5_PhiX_Lambda.fa \

sample_R2.conv.fastq > *sample*_R2.sai

where hs37d5_PhiX_lambda.fa corresponds to the hs37d5 genome sequence plus the PhiX lambda genome (control for bisulfite conversion). The resulting single-end *.sai files were paired:

bwa sampe -t 8 -T -s -P -n 0 -N 0 -r readgroupinformation \

hs37d5-PhiX_Lambda.fa sample_R1.sai sample_R2.sai \

*sample*_R1.conv.fastq *sample*_R2.conv.fastq > sample_Sampe_output

The readgroupinformation is explained above (reChIP-seq). The resulting *sample*_Sampe_output served as input to the reconversion step of methylCtools:

methylCtools bconv \--metrics {*samples*.reconversion.metrics} \

sample_Sampe_output \methylCtools_reconverted.bam

The resulting methylCtools.reconverted.bam was sorted by coordinate using samtools:

samtools view -Sbu methylCtools_reconverted.bam | \

samtools sort -o - sorted_lane_bamfile

Duplicates were marked using Picard tools version 1.61:

java -Xmx50G picard-1.61.jar MarkDuplicates I={sorted_lane_bamfile*} \

OUTPUT={samplesID.bwa.date.bam} \

TMP_DIR={TMP_DIR} \

VALIDATION_STRINGENCY=SILENT \

REMOVE_DUPLICATES=FALSE \

ASSUME_SORTED=TRUE \

CREATE_INDEX=TRUE \

MAX_RECORDS_IN_RAM=12500000 \

METRICS_FILE={samplesID.PicardMarkDupmetrics.date.txt}

### normR normalization and enrichment calling

ChIP as well as reChIP aims at enriching chromatin fragments that contains a certain antigen or combination of antigens. The identity of these chromatin fragments is determined by sequencing and after mapping to the appropriate genome they are assigned a genomic location. The accumulation of reads from a (re)ChIP library at certain genomic regions is then viewed as a proxy for the presence of a certain antigen and/or combination of antigens. Statistically, the mere accumulation of (re)ChIP reads is, however, not sufficient for this conclusion, but has to be compared with an appropriate control, that is, we are interested in the enrichment of (re)ChIP reads over the control.

To quantify enrichment, we developed the normR computational framework. normR fits a binomial mixture model, where the mixture components correspond to background B and enrichment E. Given the counts of reads in genomic bins for the control and the (re)ChIP, we maximize the likelihood:


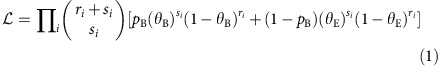


where *r*_*i*_ denotes the number of reads in the control, *s*_*i*_ in the (re)ChIP for bin *i*, *p*_B_ the mixture proportion for the background, *θ*_B_ the proportion of reads from the (re)ChIP in the background, and *θ*_E_ in enrichment given a total number of reads for a bin *r*_*i*_+*s*_*i*_.

The parameters *p*_B_, *θ*_B_ and *θ*_E_ are found by Expectation-Maximization, where we use the posterior probability that bin *i* is generated by the background (enrichment) model:


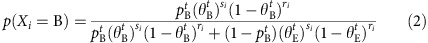


at the values of the parameters 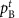, 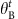 and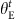 at iteration *t* to re-estimate them:


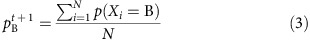


where *N* corresponds to the total number of bins; and


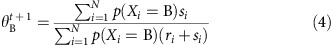



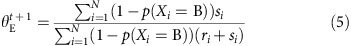


until convergence, that is, the likelihood does not change anymore (*ɛ*≤0.001). Expectation-Maximization converges to a local maximum—to increase the chance to find the global maximum, the EM is started with initial values for *θ*_B_ and *θ*_E_ set close to 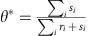.

Every bin *i* is tested for significance against the background component. Resulting P values are filtered using the T method^41^ (P value threshold 0.0001) and transformed to q values^42^. normR is available for download as Supplementary Software and at https://bioconductor.org/packages/normr.

### normR normalized and regularized log enrichment

On the basis of the normR normalization, a normalized and regularized log enrichment can be calculated. To account for noise generated by low counts, we adjusted the counts by adding pseudo counts for control and (re)ChIP, respectively. These pseudo counts were taken to be the average read counts in the background component:


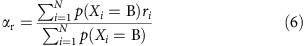



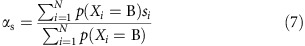


Using these pseudo counts, we calculated a regularized log enrichment


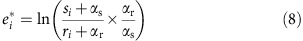


where the second term shifts *e*_i_* to zero for the background component. To account for the enrichment achieved by a ChIP experiment, we divide *e*_i_* by the log of average enrichment factor:


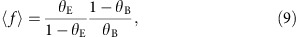


to arrive at a normalized and regularized enrichment *e*_*i*_:


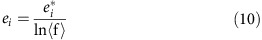


### TSS and CpG island classification

The 5′-ends of GENCODE version 19 (ref. 43) protein coding transcripts were extracted and ordered by the TSS coordinate, transcription direction, and the gene name. Transcripts with the same TSS, transcription direction and gene name were combined and the transcript with the highest number of RNA-seq reads in its first exon was chosen as a representative. This yielded 73,043 TSSs. We counted fragment centres in a window spanning ±750 base pairs from these TSSs for Input, H3K4me1, H3K4me3, H3K9me3, H3K27ac, H3K27me3, H3K36me3, H3K4me3 reChIP and H3K27me3 reChIP in R (ref. 44) using the bioconductor package bamsignals (version 1.4 https://bioconductor.org/packages/bamsignals). For CpG islands, we downloaded the CpG island annotation from UCSC and counted reads along the complete length of the CpG island.

library(bamsignals)

# Genomic Ranges object tss or CpG island

gr = tss

count = bamCount(

bampath = bampath,

gr = gr,

mapqual = 20,

paired.end = “midpoint”,

tlen.filter = c(120,240),

filteredFlag = 1024

)

The (re)ChIP libraries were normalized against the appropriate control using normR:

library(normr)

norm = enrichR(

treatment = counts.(re)ChIP,

control = counts.control,

genome = gr

)

We called a TSS or CpG island enriched in a (re)ChIP using a *q* value threshold of 0.001, 0.01 and 0.1:

enriched.0.001 = !is.na(getClasses(norm, fdr = 0.001))

enriched.0.01 = !is.na(getClasses(norm, fdr = 0.01))

enriched.0.1 = !is.na(getClasses(norm, fdr = 0.1))

This procedure yields for every TSS or CpG island and for every (re)ChIP over control a classification, whether they are enriched or not. We used the enrichment calls for H3K4me3, H3K27me3, H3K4me3 reChIP and H3K27me3 reChIP over input to cluster the TSSs or CpG islands in eight classes: (i) no enrichment in any (re)ChIP, (ii) only enrichment in H3K27me3, (iii) only enrichment in H3K4me3, (iv) enrichment in H3K4me3 and H3K27me3 but not in the two reChIPs, (v) only enrichment in both the reChIPs but no enrichment in the primary ChIPs, (vi) enrichment in H3K27me3 and both the reChIPs, (vii) enrichment in H3K4me3 and both the reChIPs and (viii) enrichment in all the four (re)ChIPs.

### RNA-seq analysis

RNA-seq reads were counted for GENCODE version 19 (ref. 43) protein coding transcripts:

library(GenomicFeatures)

chromInfoDF = read.table(‘‘hs37d5.chrom_sizes'')

chromInfoDF = data.frame(

chrom = chromInfoDF[, 1],

length = chromInfoDF[, 2],

is_circular = chromInfoDF[,1] == ‘‘MT''

)

txdb = makeTxDbFromGFF(

file = ‘‘gencode.v19.annotation.gtf'',

format = ‘‘gtf'',

dataSource = ‘‘Gencode 19'',

organism = ‘‘Homo sapiens'',

chrominfo = chromInfoDF

)

ebg = exonsBy(txdb, by = ‘‘gene'')

ebg.reduce = reduce(ebg)

The RNA-seq from our sample reads were counted using bamsignals:

library(bamsignals)

gr = unlist(ebg.reduce)

count = bamCount(

bampath = bampath,

gr = gr,

mapqual = 30,

paired.end = ‘‘filter'',

ss = TRUE

)[2,]

count = tapply(count, names(gr), sum)

In case of the RNA-seq data for the 5 independent CD4+ Central Memory T cell samples^28^ we modified the script:

count = bamCount(

bampath = bampath,

gr = gr,

mapqual = 255,

paired.end = ‘‘filter''

)

count = tapply(count, names(gr), sum)

The resulting counts were normalized using DESeq2:

library(DESeq2)

dds = DESeqDataSetFromMatrix(countData, colData = sampleData, rowRanges = ebg.reduce, design = ∼ lab)

## regularized log normalization by DESeq2

rld = rlog(dds, blind = FALSE)

where we accounted for the different labs.

Finally, we normalized for the counts for the different exon length:

## generate expression matrix

mat.expr = assay(rld)

## normalize by gene length

mat.expr = apply(mat.expr, 2, function(x) x - log(geneLength, 2) + mean(log(geneLength, 2)))

### Mapping of promoter classes to genes

To map the promoter classes to genes, we used a decision tree, where we extracted the classes for the different promoters of each gene:

gene2class = tapply(class, tss$geneID, function(x){

  if (‘‘H3K4me3-only'' %in% x)

   return(‘‘H3K4me3-only'')

  if (‘‘H3K4me3-partial'' %in% x)

   return(‘‘H3K4me3-partial'')

  if (‘‘pseudo'' %in% x)

   return(‘‘pseudo'')

  if (‘‘bivalent'' %in% x)

   return(‘‘bivalent'')

  if (‘‘H3K27me3-partial'' %in% x)

   return(‘‘H3K27me3-partial'')

  if (‘‘H3K27me3-only'' %in% x)

   return(‘‘H3K27me3-only'')

  return(‘‘unmodified'')

})

### Coefficient of variation

For each gene, we determined the expression level by retransforming the length-normalized rlog values for the RNA-seq data for five independent CD4+ Central Memory T-cell samples^28^ provided by DESeq2 (see above) to the original scale. The coefficient of variation was calculated by dividing the standard deviation by the mean.

### GO enrichment analysis

The GO term annotation was downloaded from Ensembl 74 via the biomaRt package. The enrichment of the terms GO:0032502 (developmental process), GO:0008152 (metabolic process) and GO:0002376 (immune system process) was tested using the Fisher's exact test with (i) all Gencode V19 protein-coding genes and (ii) all Gencode V19 protein-coding genes overlapping genes with bivalent promoters in human ES cells as background.

### Data availability

(re)ChIP, RNA and whole-genome bisulfite sequencing data that support the findings of this study have been deposited in the ‘European Genome-Phenome Archive' with the accession code EGAS00001001568. The RNA-seq data for human CD4^+^ central memory T cells referenced in this study are available in the ‘European Nucleotide Archive' with the accession codes ERP004883 (ref. 28).

## Additional information

**Accession codes:**(re)ChIP, RNA and whole-genome bisulfite sequencing data that support the findings of this study have been deposited in the ‘European Genome-Phenome Archive' with the accession code EGAS00001001568. The RNA-seq data for human CD4^+^ central memory T cells referenced in this study are available in the ‘European Nucleotide Archive' with the accession codes ERP004883 (ref. 28).

**How to cite this article:** Kinkley, S. *et al*. reChIP-seq reveals widespread bivalency of H3K4me3 and H3K27me3 in CD4^+^ memory T cells. *Nat. Commun.* 7:12514 doi: 10.1038/ncomms12514 (2016).

## Supplementary Material

Supplementary Information Supplementary Figures 1-16, Supplementary Tables 1-5, Supplementary Note 1 and Supplementary References

Supplementary Data 1 TSS classification

Supplementary Data 2 CpG island classification

Supplementary Software Supplementary Software normR source code. Up to date versions are available on the Bioconductor web page http://bioconductor.org/packages/normr/.
