# Supplementary material for: reChIP-seq reveals widespread bivalency of H3K4me3 and H3K27me3 in CD4+ memory T cells
Source: Nat Commun. 2016 Aug 17;7:12514. doi: 10.1038/ncomms12514 (PMC4992058; doi:10.1038/ncomms12514)
Supplement: Supplementary Information — Supplementary Figures 1-16, Supplementary Tables 1-5, Supplementary Note 1 and Supplementary References [file ncomms12514-s1.pdf]

Supplementary Information

Supplementary Figures

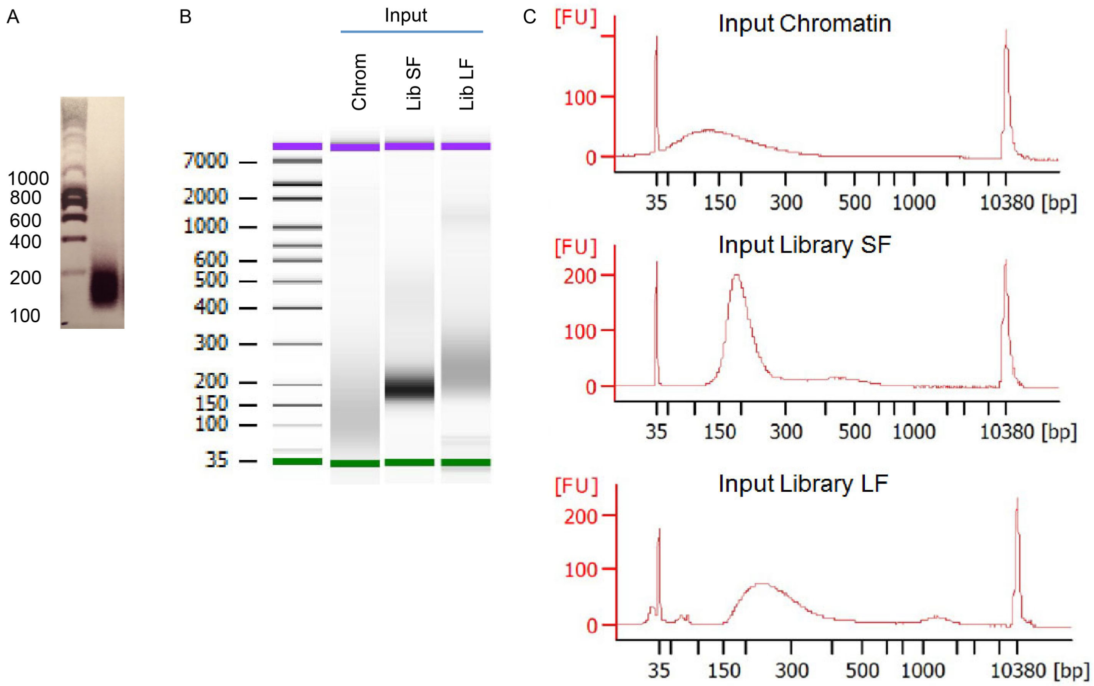

Supplementary Figure 1 Mono-/di-nucleosomal chromatin input and size selection. (A) 200ng of sheared input chromatin was run on a 1.5% agarose gel and stained Gel Red DNA stain. **(B** and **C)** 1ng of DNA sheared input chromatin and input library were run on a Bioanalyzer to estimate input and library fragment length. The input library was size selected with Ampure beads into short (SF) and large (LF) fragment sizes.

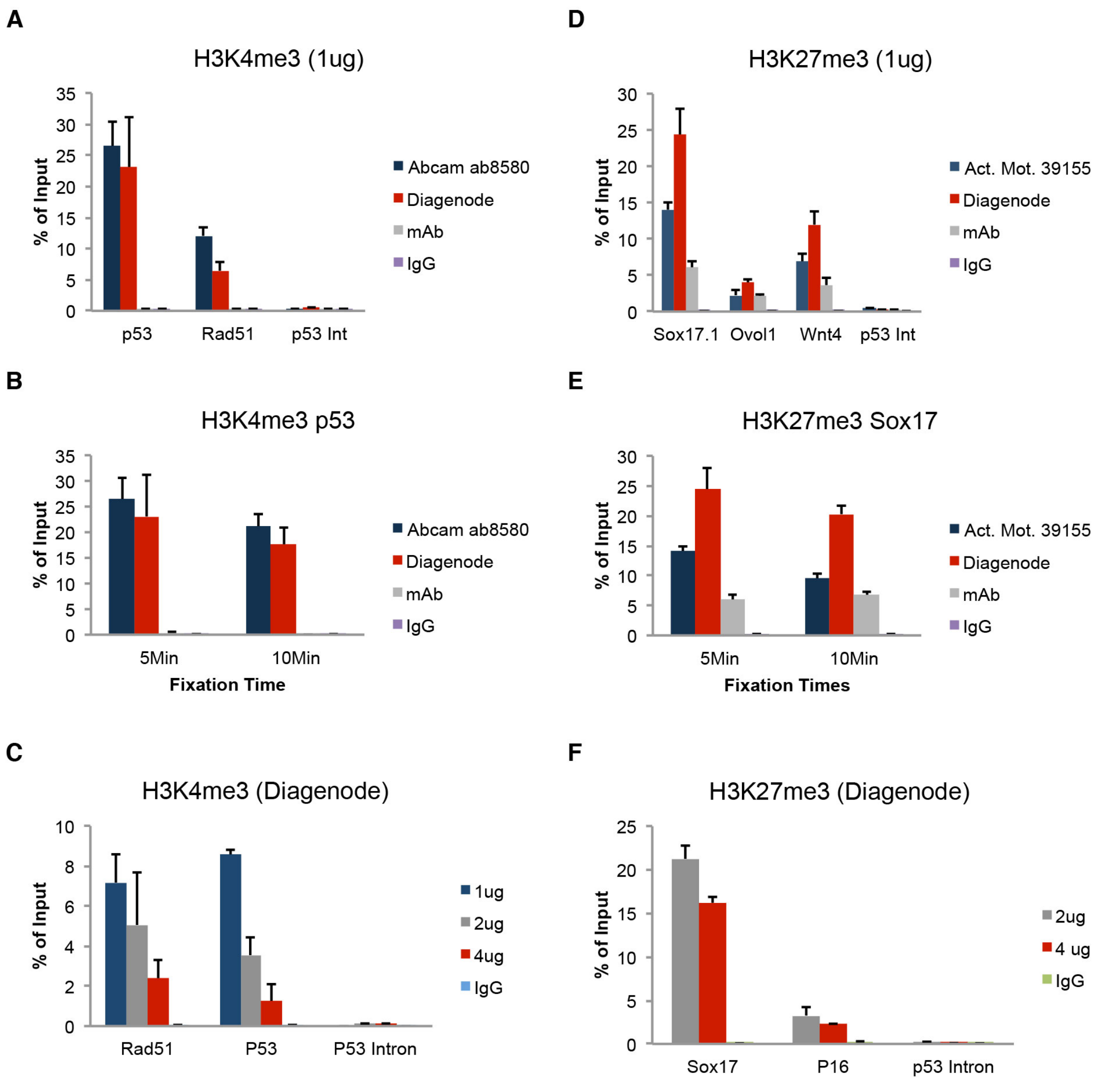

Supplementary Figure 2 Representative ChIP-qPCR analysis of H3K4me3 and H3K27me3 antibody specificity and optimization conditions. (A) and (B) Two different H3K4me3-specific antibodies were tested for their precipitation efficiency (A), and for ideal fixation time (B). (C) Varying amounts of H3K4me3-specific antibodies (Diagenode) were evaluated for their precipitation efficiency and specificity using H3K4me3 positive (Rad51, P53) and H3K4me3 negative regions (P53 intron) from 1 x 10^6 TCMs. (D) and (E) Two different H3K27me3-specific antibodies were tested for their precipitation efficiency (D), and for ideal fixation time (E). (F) Varying amounts of H3K27me3-specific antibodies (Diagenode) were evaluated for their precipitation efficiency and specificity using H3K27me3 positive (Sox17, P16) and H3K27me3 negative regions (P53 intron) from 1 x 10^6 TCMs.

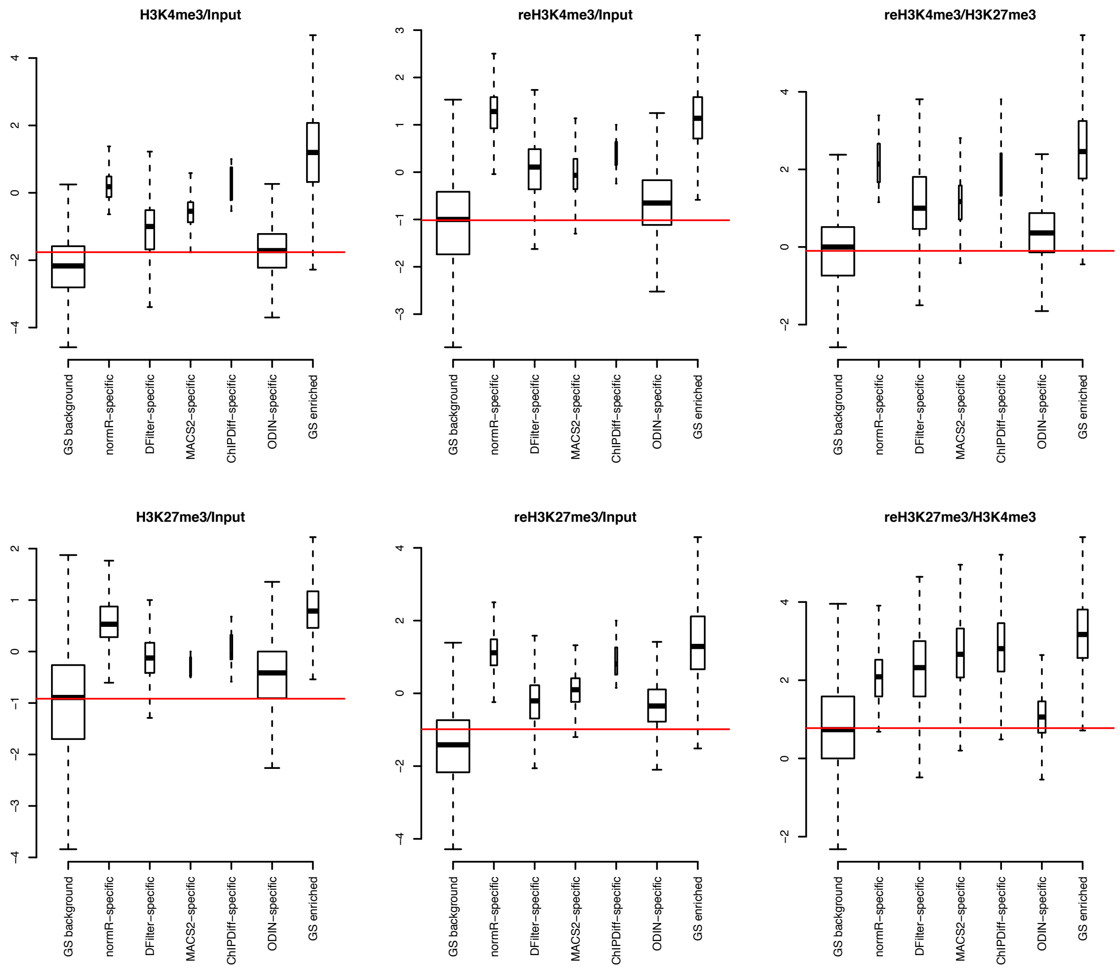

Supplementary Figure 3 $\log_2$ enrichment of (re)ChIP over control in tool-specific bins. On the y axis $\log_2$ ratios between (re)ChIP and control. The red line indicates the expected ratio by dividing the total number of reads in the (re)ChIP by the total number of reads in the control. The width of the boxes is proportional to fourth root of the number of bins in each category.

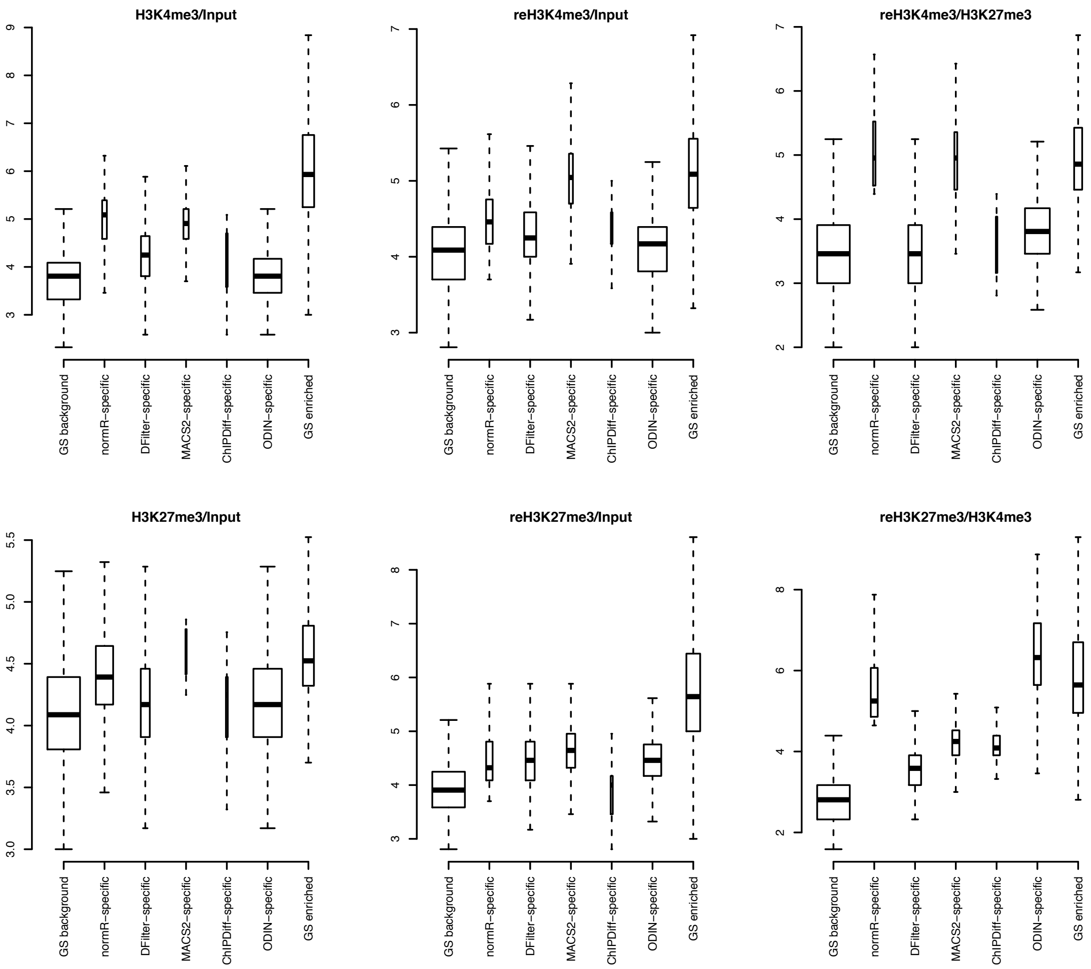

Supplementary Figure 4 log_2 (re)ChIP + control. On the y axis log_2 of the sum of (re)ChIP and control. The width of the boxes is proportional to fourth root of the number of bins in each category.

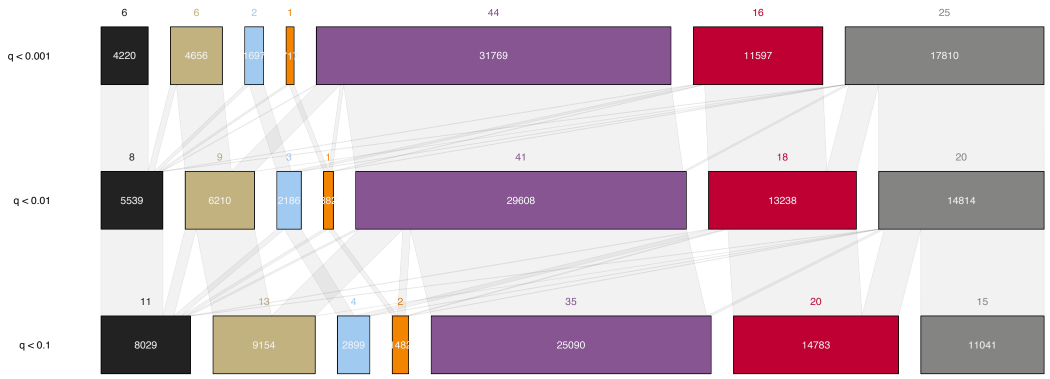

Supplementary Figure 5 TSS class assignments at different q-value thresholds. Shown are the changes in class assignments using different q-value thresholds: q < 0.001, q < 0.01 and q < 0.1. The colors indicate the class: black, bivalent; beige, H3K4me3 partial bivalent; blue, H3K27me3 partial bivalent; orange, pseudo bivalent; purple H3K4me3-only; red, H3K27me3-only; and grey, unmodified. The numbers above each box denote the percentage and the numbers within each box the frequency. The light grey quadrangles show the changes in class assignments.

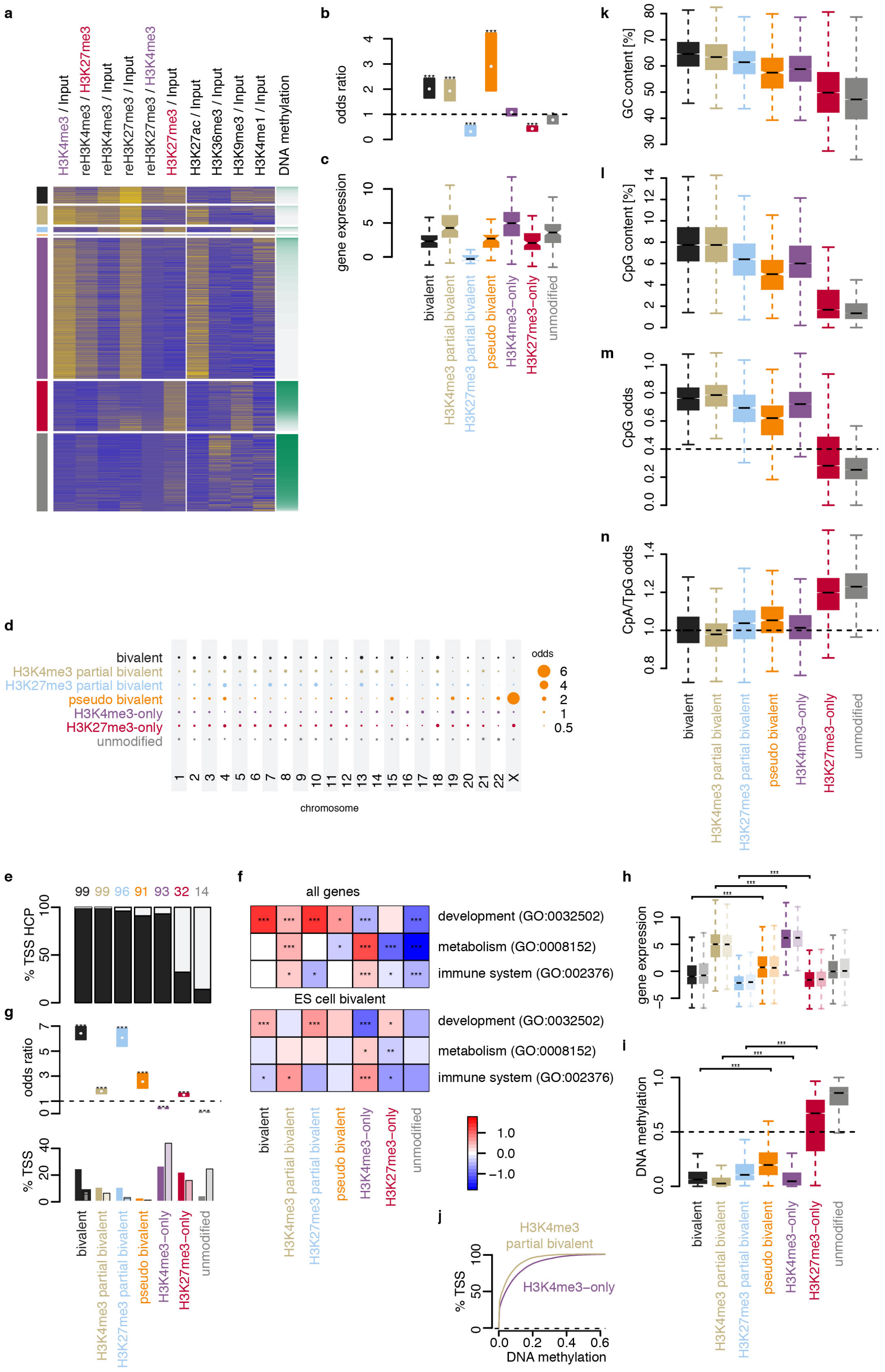

Supplementary Figure 6 All analysis at a q-value threshold q < 0.001. (a) as Fig. 2b; **(b)** as Fig. 2c. **(c)** as Fig. 2d. **(d)** as Fig. 2e. **(e)** as Fig. 3a. **(f)** as Fig. 3b. **(g)** as Fig. 3d. **(h)** as Fig. 3e. **(i)** as Fig. 3c. **(j)** as Supplementary Fig. 8. **(k)** as Fig. 4a. **(l)** as Fig. 4b. **(m)** as Fig. 4c. **(n)** as Fig. 4d.

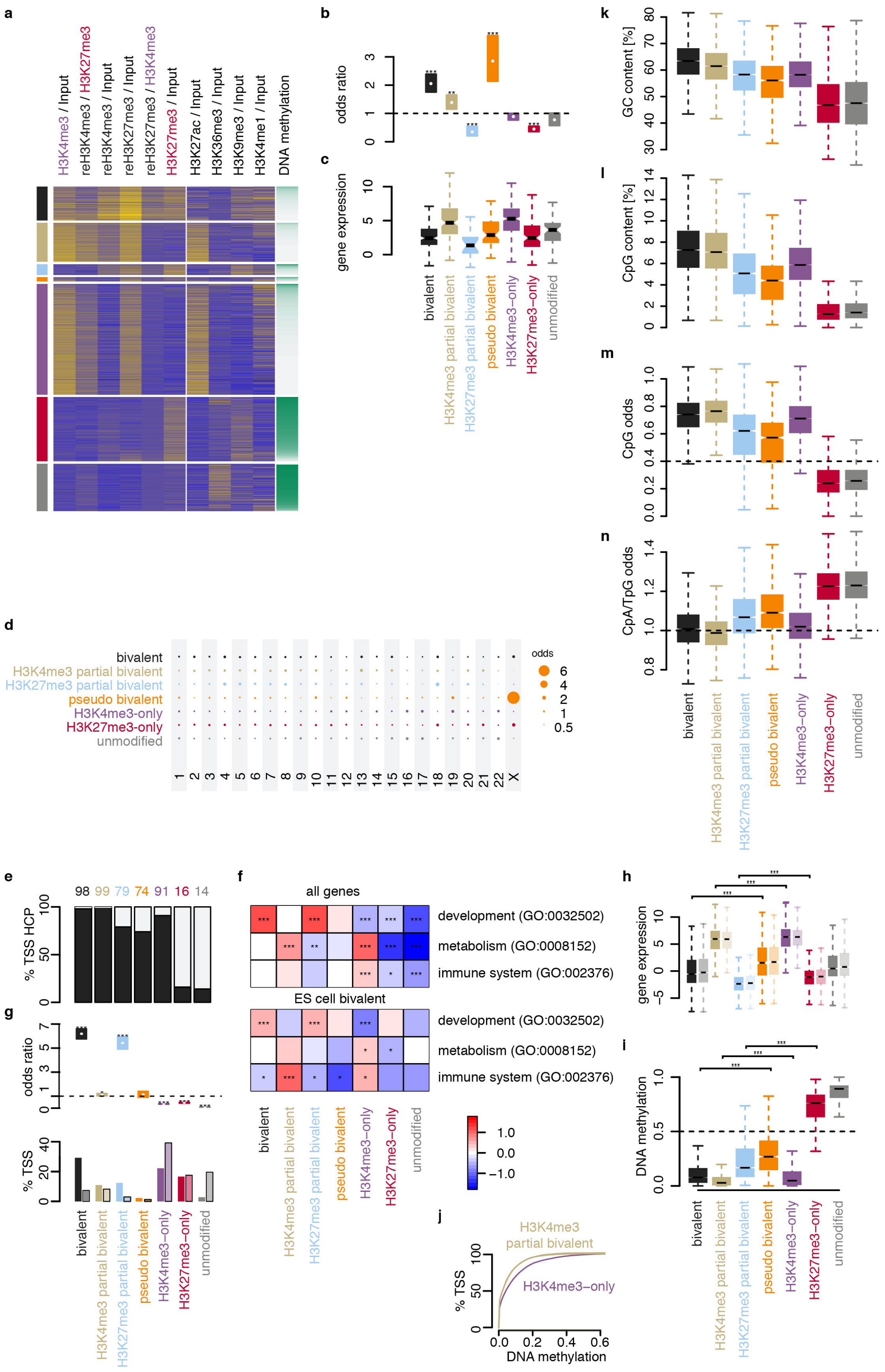

Supplementary Figure 7 All analysis at a q-value threshold q < 0.1. (a) as Fig. 2b; **(b)** as Fig. 2c. **(c)** as Fig. 2d. **(d)** as Fig. 2e. **(e)** as Fig. 3a. **(f)** as Fig. 3b. **(g)** as Fig. 3d. **(h)** as Fig. 3e. **(i)** as Fig. 3c. **(j)** as Supplementary Fig. 8. **(k)** as Fig. 4a. **(l)** as Fig. 4b. **(m)** as Fig. 4c. **(n)** as Fig. 4d.

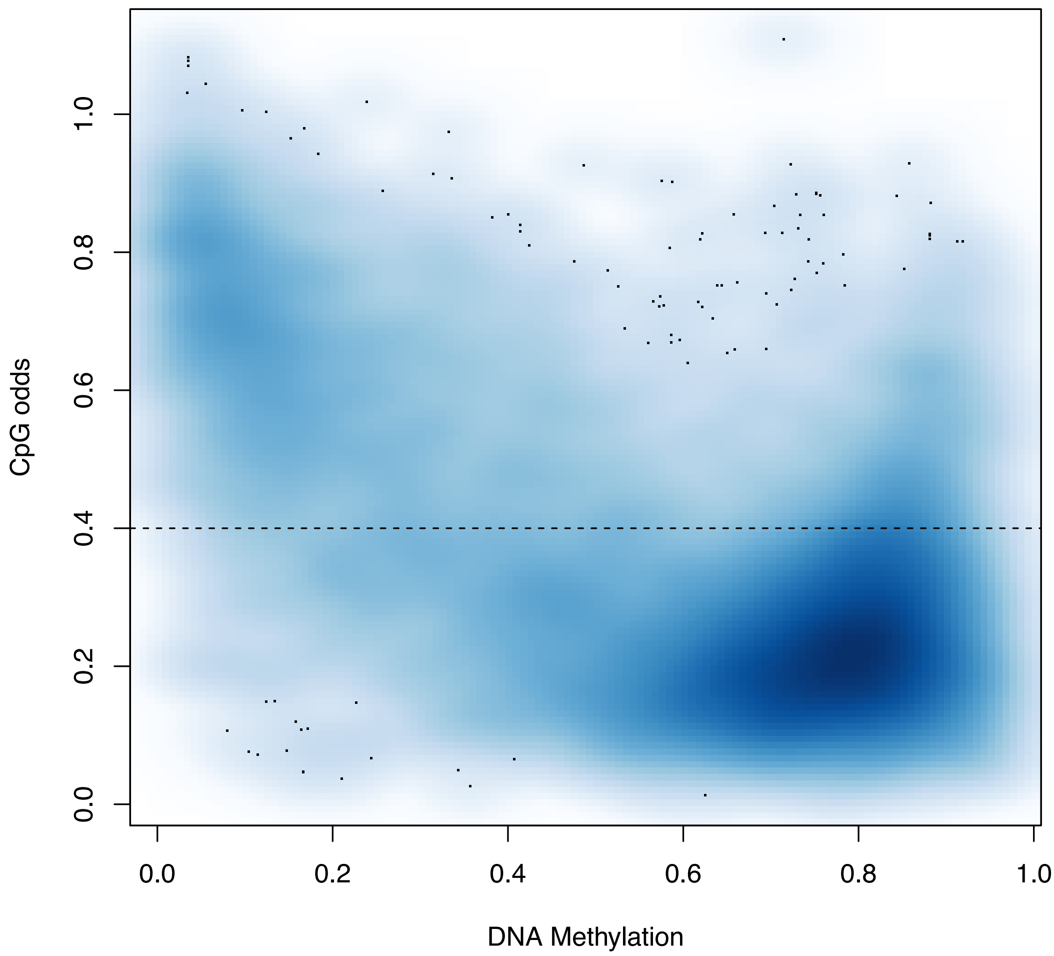

Supplementary Figure 8 DNA hypomethylated H3K27me3-only promoters are high CpG content promoters. The dashed line indicates the threshold for high CpG content promoters on the CpG odds.

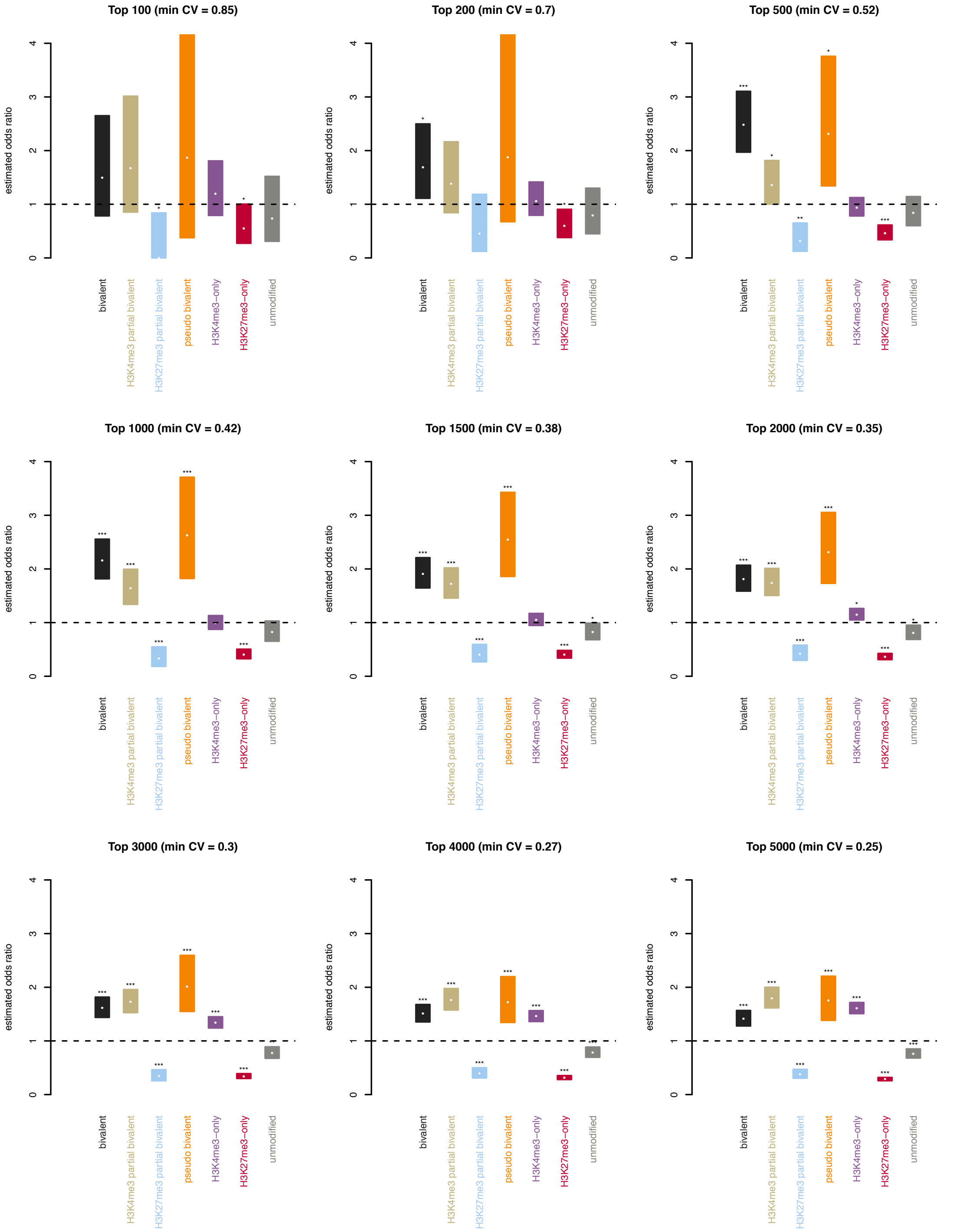

Supplementary Figure 9 Over-representation of H3K4me3 partial and pseudo bivalent genes in the most variable genes does not dependent on the choice of the top k genes.

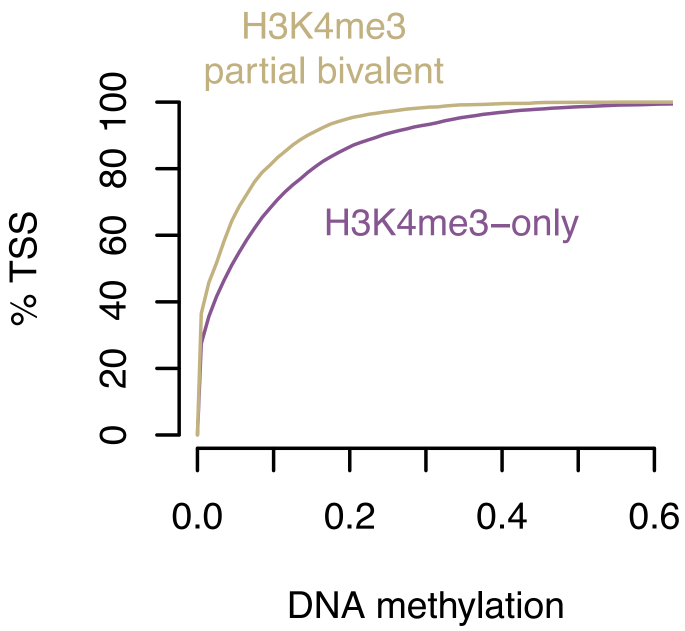

Supplementary Figure 10 H3K4me3 partial bivalent TSSs have lower DNA methylation levels than H3K4me3-only TSSs.

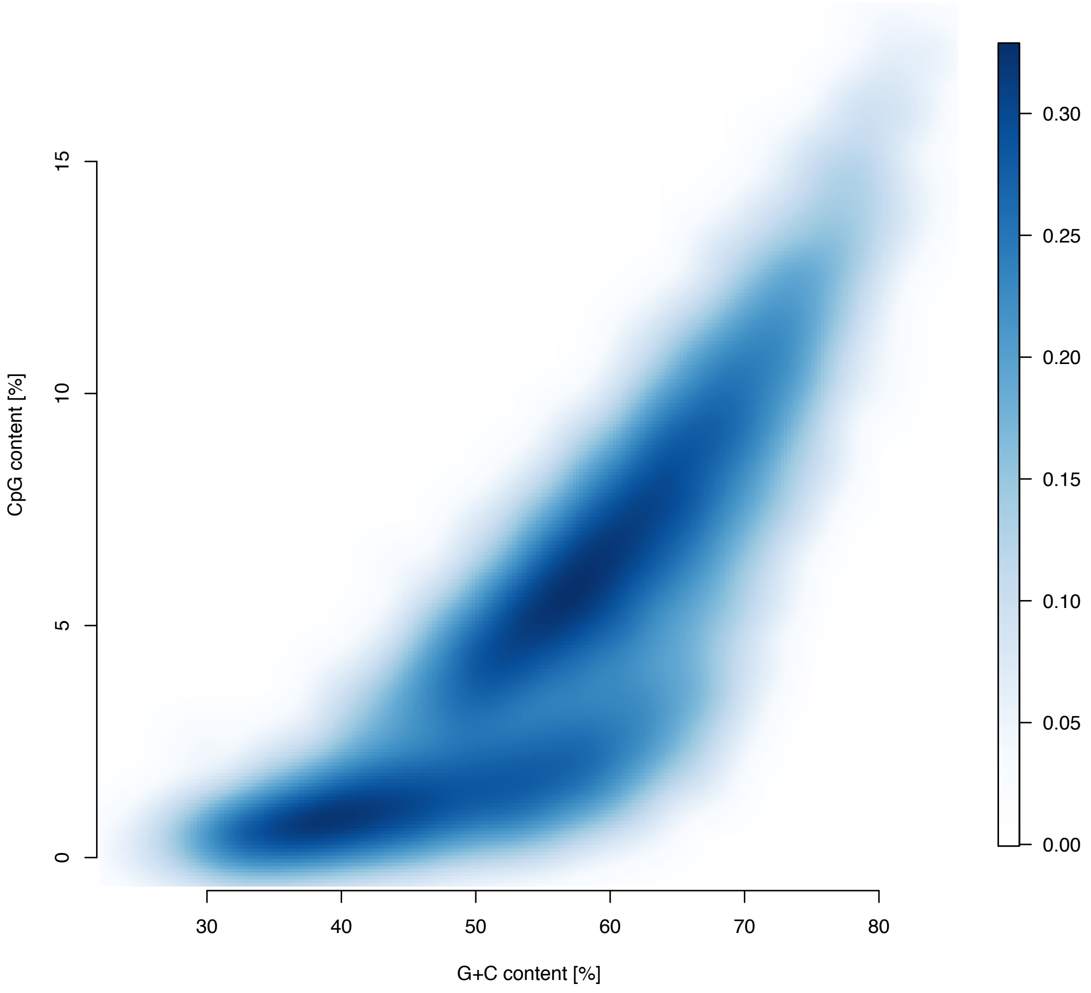

Supplementary Figure 11 Correlation between G+C and CpG content. The color indicates the density of points (see color key).

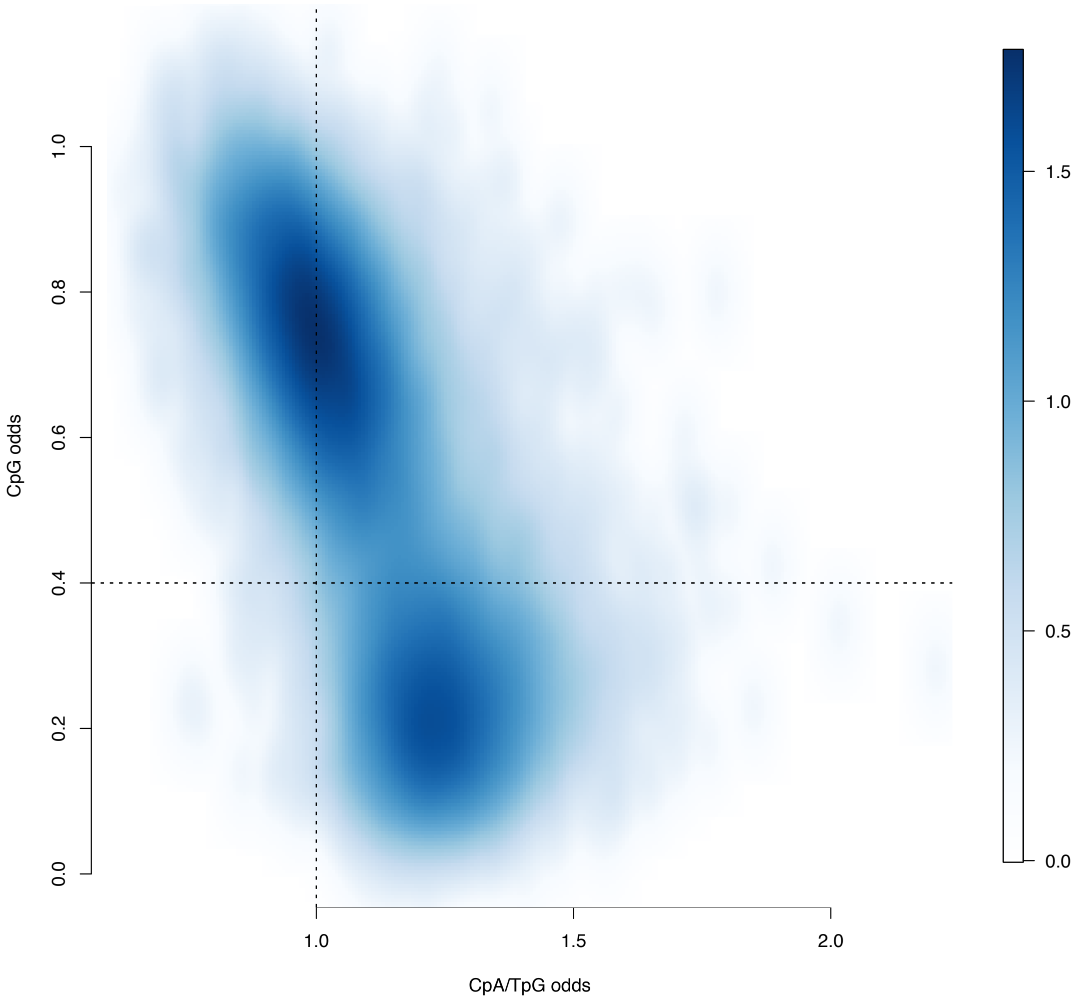

Supplementary Figure 12 Negative correlation between CpA/TpG odds and CpG odds. The color indicates the density of points (see color key).

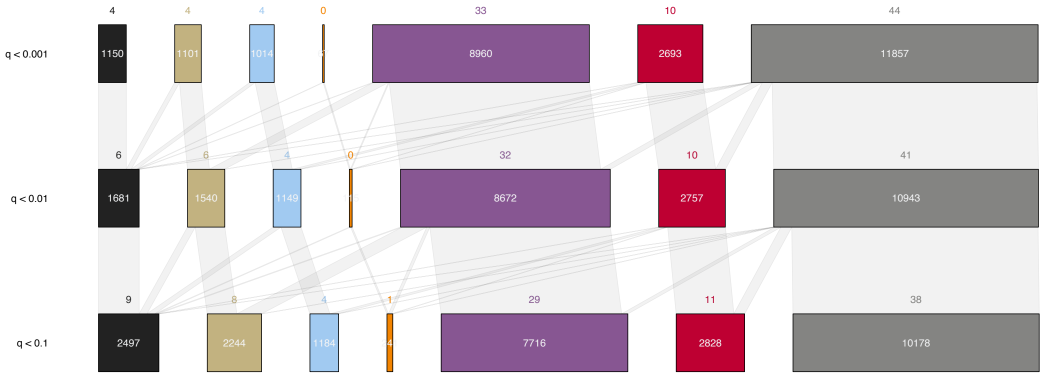

Supplementary Figure 13 CpG island class assignments at different q-value thresholds. Shown are the changes in class assignments using different q-value thresholds: q < 0.001, q < 0.01 and q < 0.1. The colors indicate the class: black, bivalent; beige, H3K4me3 partial bivalent; blue, H3K27me3 partial bivalent; orange, pseudo bivalent; purple H3K4me3-only; red, H3K27me3-only; and grey, unmodified. The numbers above each box denote the percentage and the numbers within each box the frequency. The light grey quadrangles show the changes in class assignments.

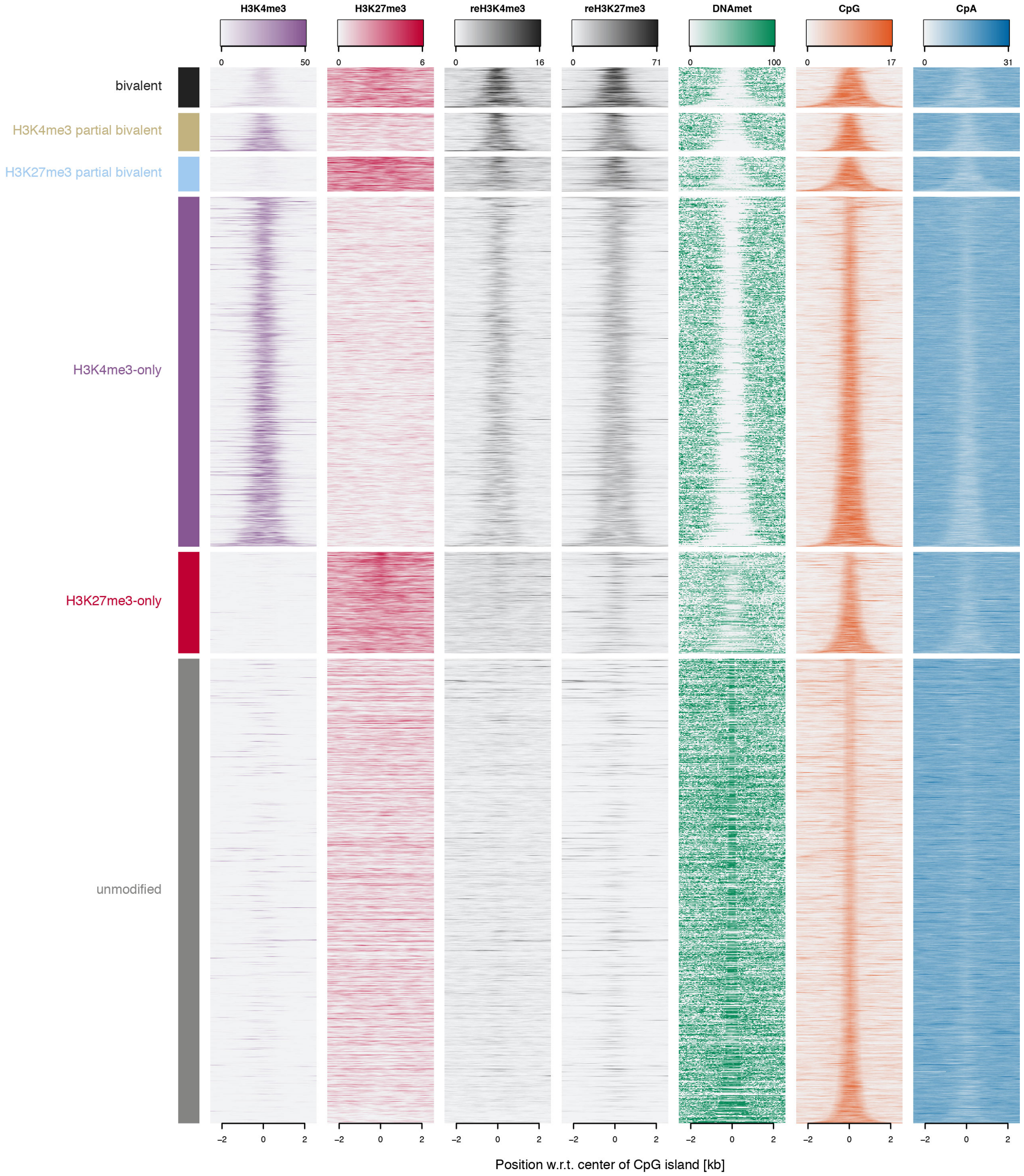

Supplementary Figure 14 CpG island states using q-value threshold q < 0.001. Same as Fig. 5.

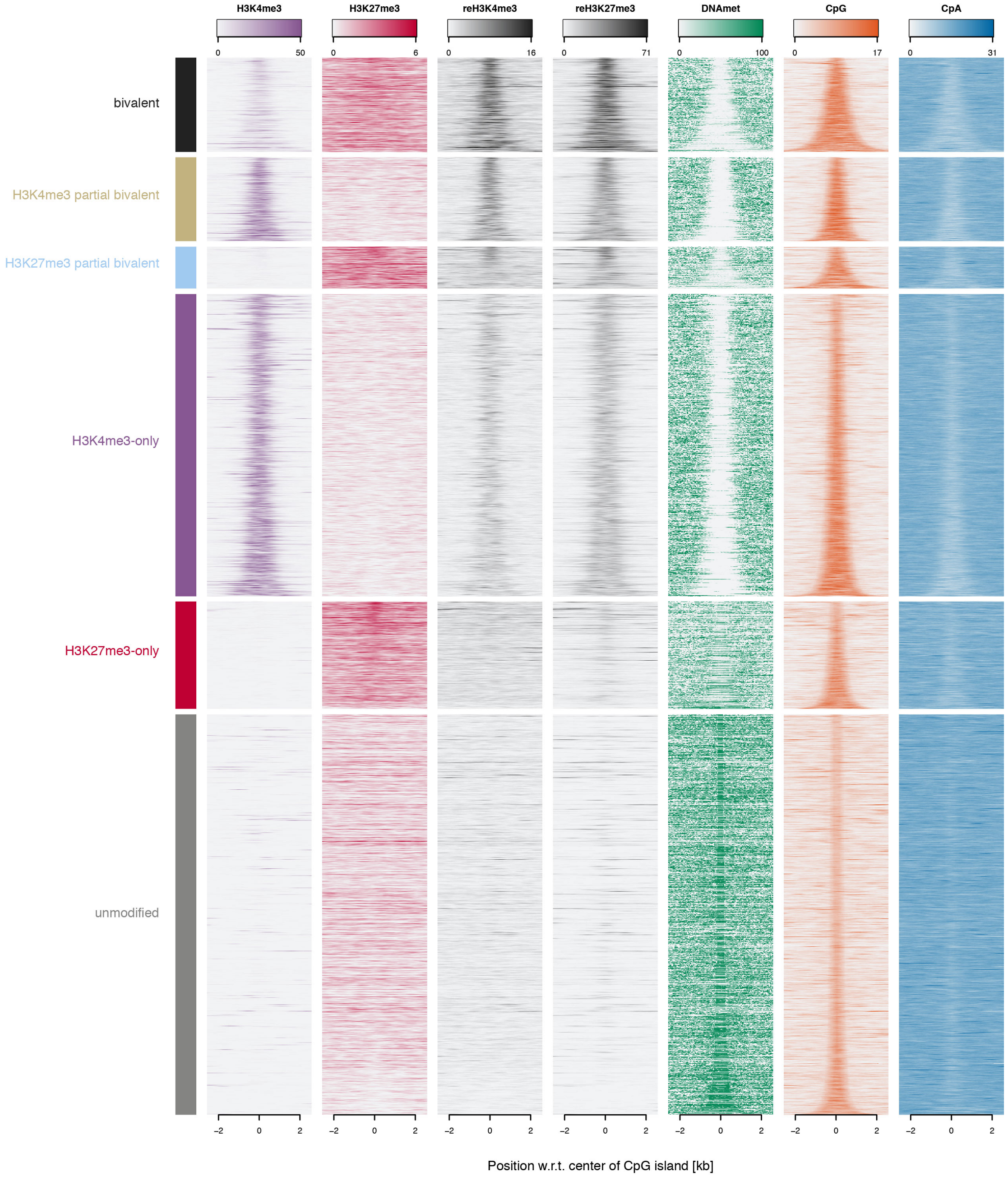

Supplementary Figure 15 CpG island states using q-value threshold q < 0.1. Same as Fig. 5.

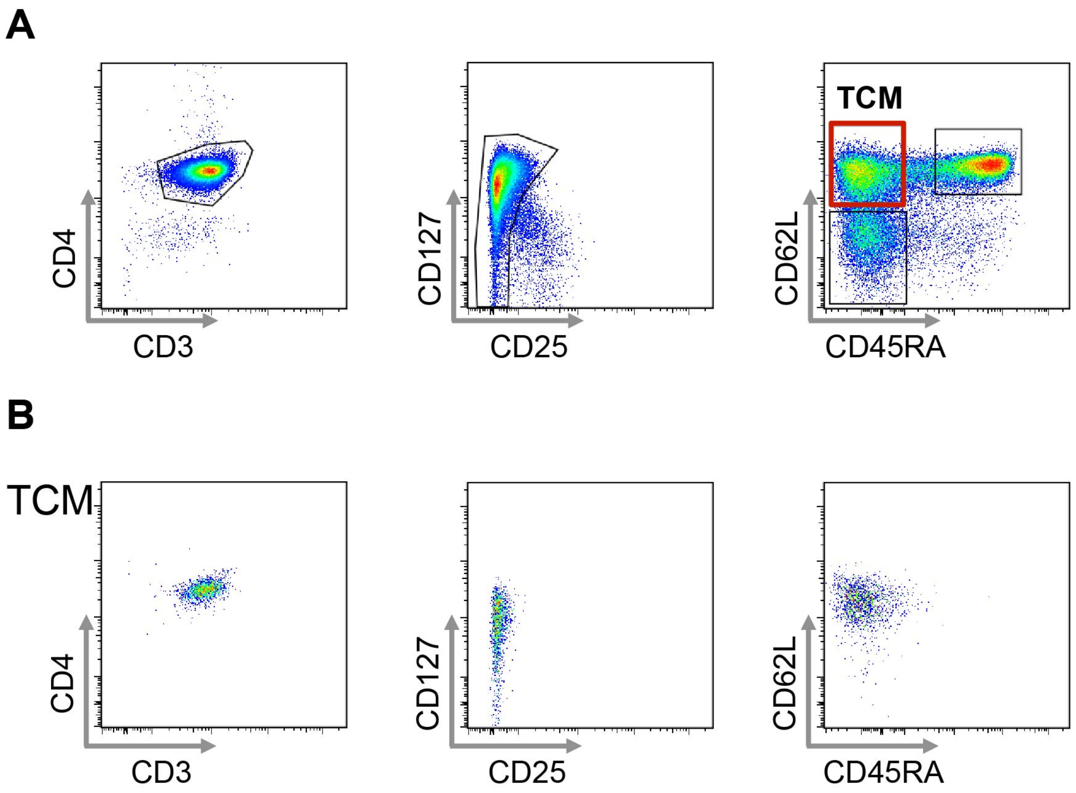

Supplementary Figure 16 Isolation strategy and purity-check of analyzed primary human central memory T cells. Peripheral blood mononuclear cells (PBMCs) were isolated from buffy coats and pre-enriched for CD4+ cells using the MACS technology. The enriched fraction was stained for surface marker expression and central memory T cells (TCM) were sorted using the indicated sorting strategy **(A)**. The sorted population was re-checked for purity before analysis **(B)**. The panel shows one representative of 3 female donors used.

Supplementary Tables

(re)ChIP	Control	normR	DFilter		MACS2		ChIPDiff	ODIN
		q < 0.01	narrow	broad	narrow	broad		q < 0.01
H3K4me3	Input	38,505	52,773	NA	43,707	NA	26,255	1,486,109
H3K27me3	Input	253,084	NA	51,809	57	1,047	1,805	1,625,179
reH3K4me3	Input	17,622	19,970	66,140	6,612	9,803	2,475	1,687,833
reH3K27me3	Input	77,548	70,241	95,431	69,088	97,391	42,929	310,069
reH3K4me3	H3K27me3	6,405	31,510	89,685	6,705	9,223	2,161	939,939
reH3K27me3	H3K4me3	27,536	25,339	75,832	21,718	30,022	23,092	20,192

Supplementary Table 1 Number of 1,000 bp bins called by each tool (3,095,689 bins total).

	normR	DFilter	MACS2	ChIPDiff	ODIN
H3K4me3/Input	0.866	0.912	0.977	0.614	1.000
H3K27me3/Input	0.978	0.824	0.024	0.041	1.000
reH3K4me3/Input	0.789	0.943	0.568	0.162	1.000
reH3K27me3/Input	0.837	0.913	0.980	0.496	1.000
reH3K4me3/H3K27me3	0.594	0.991	0.842	0.188	1.000
reH3K27me3/H3K4me3	0.865	0.936	0.952	0.700	0.536

Supplementary Table 2 Recall. Red numbers denote the best value for each row. The gold standard is based on a consensus vote by the other four tools (i.e. three or more identified this bin.

	normR	DFilter	MACS2	ChIPDiff	ODIN
H3K4me3/Input	0.895	0.635	0.787	0.996	0.023
H3K27me3/Input	0.008	0.040	0.843	0.806	0.001
reH3K4me3/Input	0.410	0.109	0.716	0.946	0.004
reH3K27me3/Input	0.853	0.694	0.680	0.995	0.214
reH3K4me3/H3K27me3	0.869	0.065	0.626	0.855	0.006
reH3K27me3/H3K4me3	0.652	0.237	0.597	0.648	0.618

Supplementary Table 3 Precision. Red numbers denote the best value for each row. The gold standard is based on a consensus vote by the other four tools (i.e. three or more identified this bin.

	normR	DFilter	MACS2	ChIPDiff	ODIN
H3K4me3/Input	0.880	0.748	0.872	0.760	0.045
H3K27me3/Input	0.016	0.077	0.048	0.077	0.003
reH3K4me3/Input	0.539	0.196	0.634	0.276	0.009
reH3K27me3/Input	0.845	0.788	0.803	0.663	0.353
reH3K4me3/H3K27me3	0.706	0.122	0.718	0.308	0.012
reH3K27me3/H3K4me3	0.743	0.378	0.734	0.673	0.574

Supplementary Table 4 F1-score. Red numbers denote the best value for each row. The gold standard is based on a consensus vote by the other four tools (i.e. three or more identified this bin.

Mouse Gene	Human Gene	TCM state	Subset
**Eomes**	EOMES	bivalent	Th1
**Tbx21**	TBX21	H3K4me3-only	Th1
**Pparg**	PPARG	pseudo bivalent	Th2
**Asb2**	ASB2	H3K4me3-only	Th2
**Gata3**	GATA3	H3K4me3-only	Th2
**Rorc**	RORC	H3K4me3-only	Th17
**Rora**	RORA	H3K4me3-only	Th17
**Nr4a3**	NR4A3	bivalent	Treg
**Foxp3**	FOXP3	H3K27me3-only	Treg
**Tgif1**	TGIF1	H3K4me3-only	Treg
**Rel**	REL	H3K4me3 partial bivalent	Treg
**Nr4a1**	NR4A1	H3K4me3-only	Treg
**Pou2f2**	POU2F2	H3K4me3-only	Treg
**Relb**	RELB	H3K4me3-only	nTreg
**Ikzf2**	IKZF2	H3K27me3 partial bivalent	nTReg
**Myb**	MYB	H3K4me3-only	iTreg
**Irf4**	IRF4	H3K4me3 partial bivalent	iTreg
**Irf8**	IRF8	H3K4me3-only	iTreg
**Fosl1**	FOSL1	bivalent	iTreg

Supplementary Table 5 T helper cell subset-specific regulators are mostly active. Shown is the gene state for T helper cell subset-specific regulators as defined by Wei *et al.* 2009[1].

Supplementary Note 1

normR works with arbitrarily defined windows (even of different size), while existing methods work genome wide and do not provide the flexibility of normR. So normR is especially suited to work on the level of windows centered around the TSS but also on the level of different-sized CpG islands. Nonetheless we compared normR to DFilter[2], MACS2[3,4], ChIPDiff[5] and ODIN[6].

To get an unbiased view, we preprocessed the input BAM files such that every tool actually uses the same alignments, i.e. we removed duplicated fragments (-F 1024), kept only reads mapped in a proper pair (-f 2) and alignments with a mapping quality higher than 20 (-q 20).

```
> samtools view -q 20 -F 1024 -f 2 <bamFile>
```

Finally, we kept only fragments longer than 120 and shorter than 240 base pairs by piping the output of samtools to a custom perl script:

```
my $tlenMin = 120;
my $tlenMax = 240;
while(<stdin>){

      my ($qID, $flag, $chr, $start, $mapq, $cigar, $mref, $mpos, $tlen)
            = split(/\t/, $_);
      $tlen = abs($tlen)
      if ($tlen > $tlenMin && $tlen < $tlenMax){
            print $_
      }
}
```

For ChIPDiff, we took only the first in pair read of proper pairs (-f 66) and converted to tag format, i.e. chr | 5' position of read | strand

```
> samtools view -q 20 -F 1024 -f 66 <bamFile>
```

"DFilter-narrow" (version 1.6) was executed using this command:

```
> <PATH_TO_DFILTER>/run_dfilter.sh \
-d=<(re)ChIP.bam> \
-c=<control.bam>  \
-o=<result.bed>   \
-f=bam            \
-bs=100           \
-ks=100           \
-lpval=6
```

and "DFilter-broad":

```
> <PATH_TO_DFILTER>/run_dfilter.sh \
-d=<(re)ChIP.bam> \
-c=<control.bam>  \
```

```
-o=<result_broad.bed>   \
-f=bam            \
-bs=100           \
-ks=100           \
-lpval=3          \
-nonzero
```

"MACS2-narrow" (version 2.1.1.20160309) was executed:

```
> <PATH_TO_MACS2>/macs2 callpeak \
-t <(re)ChIP.bam>         \
-c <control.bam>          \
-f BAMPE                  \
-g hs                     \
—n <result>
```

and "MACS2-broad"

```
> <PATH_TO_MACS2>/macs2 callpeak \
      --broad                    \
-t <(re)ChIP.bam>         \
-c <control.bam>          \
-f BAMPE                  \
-g hs                     \
—n <result_broad>
```

ChIPDiff (source code last updated March 27 2008) was executed:

```
> <PATH_TO_ChIPDiff>/ChIPDiff \
<control.tag>           \
<(re)ChIP.tag>          \
hs37d5_chromSizes       \
config.txt              \
<result>
```

The file hs37d5_chromSizes contained the chromosome identifiers and the chromosome length. The file config.txt contained following entries:

```
maxIterationNum   500
minP  0.95
maxTrainingSeqNum 10000
minFoldChage      3.0
minRegionDist     1000
```

ODIN (version 0.4.1) was executed:

```
> <PATH_TO_ODIN>rgt-ODIN      \
      -m -v                   \
      --output-dir="<result>" \
      <control.bam>           \
```

```
<(re)ChIP.bam>          \
      hs37d5_chromSizes
```

We extracted the regions that were enriched in <(re)ChIP.bam> over the control using:

```
<PATH_TO_ODIN>split-ODIN.sh <result>-diffpeaks.bed
```

which resulted in two files: <result>-diffpeaks.bed-gain.bed (enriched in the control) and <result>-diffpeaks.bed-lose.bed (enriched in (re)ChIP). We took the latter and filtered for a q-value threshold of 0.01:

```
<PATH_TO_ODIN>filter-ODIN.sh <result>-diffpeaks.bed-lose.bed 2
```

For the normR analysis we counted the fragments using bamsignals (version 1.5) within R (version 3.2.0):

```
bamFiles = list(
      Input      = "Input.bam",
      H3K4me3    = "H3K4me3.bam",
      H3K27me3   = "H3K27me3.bam",
      reH3K4me3  = "reH3K4me3.bam",
      reH3K27me3 = "reH3K27me3.bam"
)

# load Rsamtools
library(Rsamtools)
bf = BamFile(bamFiles$Input)

# restrict analysis to major autosomes and the X
chr = c(1:22, "X")

# extract seqinfo from bam Header
seqInfo = seqinfo(bf)
seqLength = seqlengths(seqInfo)[seqnames(seqInfo) %in% chr]

# define a Granges object covering the autosomes and the X
gr = GRanges(
seqnames = names(seqLength),
ranges = IRanges(start = 1, end = seqLength
)

# load bamsignals
library(bamsignals)

# count fragment midpoints in non-overlapping 1,000 bp bins
counts = lapply(
```

```
bamFiles,
      function(bampath){
            mclapply(
                  gr,
                  function(g)
                        bamProfile(
                              bampath,
                              gr = g,
                              binsize = 1000,
                              paired.ebd = "midpoint"
                        ),
                  mc.cores = 23
            )
      }
)

genome.counts = sapply(
counts,
function(x)
            unlist(
                  sapply(
                        x,
                        function(y)
                              y@signals
                  )
            )
)
```

We defined a GRanges object containing the positions of the 1,000 base pair bins:

```
# the number of bins per chromosomes
nbins = as.integer((seqLength – 1) / 1000) + 1
names(nbins) = chr

# allocate memory for the start and end positions
starts = vector(mode="integer", length = sum(nbins))
ends = vector(mode = "integer", length = sum(nbins))

off = 0
for(ch in chr){
      starts[1:nbins[ch] + off] = seq(1,seqLength[ch], by = 1000)
      ends[1:nbins[ch] + off] = starts[1:nbins[ch] + off] + 999
      # make sure that no bin lies outside the reference coordinates
```

```
        ends[nbins[ch] + off] = seqLength[ch]
        off = off + nbins[ch]
}

bins = Granges(
seqnames = Rle(chr, nbins),
ranges = IRanges(
        start = starts,
        end = ends
)
)
```

Then we added the fragment counts as a data .frame to the bins object

```
bins$counts = genome.counts
```

and performed the normR analysis

```
analyze = list(
        # H3K4me3 against Input
        H3K4me3 = c("H3K4me3", "Input"),
        # H3K27me3 against Input
        H3K27me3 = c("H3K27me3", "Input"),
        # reH3K4me3 against Input
        reH3K4me3_Input = c("reH3K4me3", "Input"),
        # reH3K27me3 against Input
        reH3K27me3_Input = c("reH3K27me3", "Input"),
        # reH3K4me3 against H3K27me3
        reH3K4me3_H3K27me3 = c("reH3K4me3", "H3K27me3"),
        # reH3K27me3 against H3K4me3
        reH3K27me3_H3K4me3 = c("reH3K27me3", "H3K4me3")
)

library(normr)
genome.norm = lapply(
        analyze,
        function(what)
                enrichR(
                        treatment = bins$counts[, what[1]],
                        control = bins$counts[, what[2]],
                        genome = bins
                )
)
```

```
# get the enriched bins at a q-value threshold of 0.01, i.e 1% FDR
enriched = sapply(
      genome.norm,
      function(x)
        as.integer(getQvalues(x) < 0.01 & is.finite(getQvalues(x)))
)
```

To compare the results we mapped the peak locations found by the other tools to the 1,000 base pair bins:

```
# get the chrom, start and end of a peak
chrom = <chr> peak caller X
start = <start> start of identified peak
end = <end> end of identified peak

# remove the peaks not on chromosomes 1 – 22 or X
start = start[chrom %in% chr]
end = end[chrom %in% chr]
chrom = chrom[chrom %in% chr]

# define GRanges object with the peaks
peaks = GRanges(
      seqnames = chrom,
      IRanges(start = start, end = end)
)

# check overlap with the 1,000 base pair bins
ovl = findOverlaps(bins, peaks)
res = rep(0, length(bins))
res[queryHits(ovl)] = 1
```

This results in 1,000 base pair bins called enriched or overlapping a peak (Supplementary Table 1). In most cases ODIN calls way more regions than all the other tools. Next we evaluated the "performance" of each tool by defining a gold standard, based on the other tools: i.e. we called a bin enriched/positive if at least three out of the four other tools identified this bin. On the basis of recall (Supplementary Table 2) ODIN is the best tool.

On the basis of precision (Supplementary Table 3) ChIPDiff performs the best, while ODIN seems to call a lot of regions that are ODIN-specific. Of note ODIN is designed to call differences between samples/conditions of the same ChIP-seq target, so it may not be suited for the task to find enrichment.

Finally, we checked the F1-score, which corresponds to a weighted average of precision and recall (Supplementary Table 4). Here, in half of the cases normR performs best. This whole analysis hinges on the definition of a gold standard.

We believe that enrichment/peak calling is an ill-defined problem as we do not know the ground truth. Taking e.g. the consensus vote from other tools as a gold standard does not address the problem that the other tools may fail to find the relevant regions. For example in H3K27me3 normR finds many more regions than any of the other tools. This leads to a high recall (98%), i.e. most of the regions called by at least three other tools have also been called by normR, but leads to a low precision (< 1%), because most of the regions called by normR are normR-specific.

The question is: are these normR-specific regions genuine H3K27me3 enriched regions or are they false positives as suggested from the other tools? To answer this question, bins were called enriched if they were called by at least three out of the five tools – defining a gold standard. We extracted for each tool those bins that were called by this tool but were not enriched according to the gold standard, i.e. tool-specific bins. If the tool-specific bins were false positives, we expect that these bins should show lower than the gold standard bins or no enrichment in H3K27me3. Alternatively, if these are genuine, we expect high H3K27me3 enrichment. Thus, we calculated an unnormalized log2 ratio between the counts for H3K27me3 ChIP and for Input fragments and compared this "log enrichment" for the tool-specific bins. This revealed that normR-specific bins showed the highest enrichment (Supplementary Fig. 3), except for the enrichment of reH3K27me3 over H3K4me3. These enrichments are comparable to the enrichment in gold standard enriched bins, while the other tool-specific enrichments are much closer to the background, indicating that normR-specific bins are more likely to be really enriched compared to other tool-specific bins.

The reason for the lower enrichment seen in reH3K27me3 over H3K4me3 seems to be that although the other tools (except of ODIN) call bins with a higher $\log_2$ enrichment, the total coverage of reH3K27me3 + H3K4me3 in the bins specific for the other tools is much lower: between 8 and 16 fragments (Supplementary Fig. 4). Our statistical treatment removes low coverage regions as they cannot attain low p-values in a binomial test to begin with, thus they failed detection by normR because there is no statistical ground to call them enriched.

Taken together our results show that normR performs comparable to other tools. On the level of observed enrichments it actually outperforms the other tools in detecting highly enriched bins. Finally, normRs' flexibility in the assignment of bins allows for focusing the analysis on relevant regions such as TSSs and CpG islands – a feature that is not available in the other tools.

Supplementary References

1. Wei, G. *et al.* Global mapping of H3K4me3 and H3K27me3 reveals specificity and plasticity in lineage fate determination of differentiating CD4+ T cells. *Immunity* **30,** 155–167 (2009).
2. Kumar, V. *et al.* Uniform, optimal signal processing of mapped deep-sequencing data. *Nat. Biotechnol.* **31,** 615–622 (2013).
3. Zhang, Y. *et al.* Model-based Analysis of ChIP-Seq (MACS). *Genome Biol.* **9,** R137 (2008).
4. Feng, J., Liu, T. & Zhang, Y. Using MACS to identify peaks from ChIP-Seq data. *Curr Protoc Bioinformatics* **Chapter 2,** Unit 2.14 (2011).
5. Xu, H., Wei, C.-L., Lin, F. & Sung, W.-K. An HMM approach to genome-wide

identification of differential histone modification sites from ChIP-seq data. *Bioinformatics* **24,** 2344–2349 (2008).
6. Allhoff, M. *et al.* Detecting differential peaks in ChIP-seq signals with ODIN. *Bioinformatics* **30,** 3467–3475 (2014).
