# Supplementary material for: reChIP-seq reveals widespread bivalency of H3K4me3 and H3K27me3 in CD4+ memory T cells
Source: Nat Commun. 2016 Aug 17;7:12514. doi: 10.1038/ncomms12514 (PMC4992058; doi:10.1038/ncomms12514)
Supplement: Supplementary Software — normR source code. Up to date versions are available on the Bioconductor web page http://bioconductor.org/packages/normr/. [file ncomms12514-s4.zip › Supplementary Software/normr_0.99.1.tar.gz!/normr/inst/doc/normr.html]

normr: normR obeys mixtures Rules - Normalization & Difference Calling in ChIP-seq Data


# normr: normR obeys mixtures Rules - Normalization & Difference Calling in ChIP-seq Data

#### *Johannes Helmuth*

#### *2016-02-10*

# Contents

- 1 Introduction to normr
  - 1.1 Loading some toy data
  - 1.2 Calling Enrichment with an Input Control
    - 1.2.1 Giving bampath
    - 1.2.2 Writing output
  - 1.3 Calling Differential Enrichment between two Conditions
    - 1.3.1 Writing output
  - 1.4 Identify Regimes of Enrichment
    - 1.4.1 Writing output

# 1 Introduction to normr

```
#knitr code
plot(1:10)
plot(10:1)
```

Figure caption

## 1.1 Loading some toy data

We have load some libraries

## 1.2 Calling Enrichment with an Input Control

### 1.2.1 Giving bampath

### 1.2.2 Writing output

## 1.3 Calling Differential Enrichment between two Conditions

### 1.3.1 Writing output

## 1.4 Identify Regimes of Enrichment

Use counts from normr object in Enrichment Call

### 1.4.1 Writing output
